# Functional characterization of the 19q12 amplicon in grade III breast cancers

**DOI:** 10.1186/bcr3154

**Published:** 2012-03-20

**Authors:** Rachael Natrajan, Alan Mackay, Paul M Wilkerson, Maryou B Lambros, Daniel Wetterskog, Monica Arnedos, Kai-Keen Shiu, Felipe C Geyer, Anita Langerød, Bas Kreike, Fabien Reyal, Hugo M Horlings, Marc J van de Vijver, Jose Palacios, Britta Weigelt, Jorge S Reis-Filho

**Affiliations:** 1 The Breakthrough Breast Cancer Research Centre, The Institute of Cancer Research, 237 Fulham Road, London, SW3 6JB, UK; 2 Department of Genetics, Institute for Cancer Research, Norwegian Radium Hospital, Oslo University Hospital, Ullernchausèen 70, Montebello, Oslo, 0310, Norway; 3 Institute for Radiation Oncology Arnhem, Wagnerlaan 47, Arnhem 6815 AD, The Netherlands; 4 Department of Surgery, Institut Curie, 26 rue d'Ulm, Paris, 75005, France; 5 Department of Pathology, Academic Medical Center, Meibergdreef 9, Amsterdam, 1105 AZ, The Netherlands; 6 Servicio de Anatomia Patologica, HHUU Virgen del Rocío, Avda. Manuel Siurot, s/n, Seville, 41013, Spain; 7 Signal Transduction Laboratory, Cancer Research UK London Research Institute, 44 Lincoln's Inn Fields, London WC2A 3LY, UK

## Abstract

**Introduction:**

The 19q12 locus is amplified in a subgroup of oestrogen receptor (ER)-negative grade III breast cancers. This amplicon comprises nine genes, including cyclin E1 (*CCNE1*), which has been proposed as its 'driver'. The aim of this study was to identify the genes within the 19q12 amplicon whose expression is required for the survival of cancer cells harbouring their amplification.

**Methods:**

We investigated the presence of 19q12 amplification in a series of 313 frozen primary breast cancers and 56 breast cancer cell lines using microarray comparative genomic hybridisation (aCGH). The nine genes mapping to the smallest region of amplification on 19q12 were silenced using RNA interference in phenotypically matched breast cancer cell lines with (MDA-MB-157 and HCC1569) and without (Hs578T, MCF7, MDA-MB-231, ZR75.1, JIMT1 and BT474) amplification of this locus. Genes whose silencing was selectively lethal in amplified cells were taken forward for further validation. The effects of cyclin-dependent kinase 2 (CDK2) silencing and chemical inhibition were tested in cancer cells with and without *CCNE1 *amplification.

**Results:**

19q12 amplification was identified in 7.8% of ER-negative grade III breast cancer. Of the nine genes mapping to this amplicon, *UQCRFS1*, *POP4*, *PLEKHF1*, *C19ORF12*, *CCNE1 *and *C19ORF2 *were significantly over-expressed when amplified in primary breast cancers and/or breast cancer cell lines. Silencing of *POP4, PLEKHF1, CCNE1 *and *TSZH3 *selectively reduced cell viability in cancer cells harbouring their amplification. Cancer cells with *CCNE1 *amplification were shown to be dependent on CDK2 expression and kinase activity for their survival.

**Conclusions:**

The 19q12 amplicon may harbour more than a single 'driver', given that expression of POP4, PLEKHF1, CCNE1 and TSZH3 is required for the survival of cancer cells displaying their amplification. The observation that cancer cells harbouring *CCNE1 *gene amplification are sensitive to CDK2 inhibitors provides a rationale for the testing of these chemical inhibitors in a subgroup of patients with ER-negative grade III breast cancers.

## Introduction

Breast cancer is a heterogeneous disease encompassing a number of distinct entities, characterised by distinct biological features and clinical behaviour. Recent evidence has suggested that this heterogeneity is underpinned by distinct patterns of genomic aberrations, which may contribute to the different transcriptomic profiles and clinical phenotypes [1-4]. Importantly, it has been shown that oestrogen receptor (ER)-positive and -negative breast cancers are fundamentally different diseases, with distinct transcriptomic profiles, gene copy number aberrations and somatic structural rearrangements [5-8].

Based on the concept of oncogene addiction, we [9,10] and others [11,12] have demonstrated that the constellation of genes that are consistently overexpressed when amplified is enriched for genes selectively required for the survival of cancer cells harbouring their amplification and can be exploited as potential therapeutic targets. Using a combination of microarray-based comparative genomic hybridisation (aCGH) and gene expression profiling [11-19], previous studies have identified genes which are consistently overexpressed when amplified and suggested potential "amplicon drivers" (for example, *FGFR1*, *FGFR2*, *GAB2*, *PPAPDC1B *and *ZNF703*). It should be noted, however, that whilst many potential targets have been postulated, critical molecular drivers of several amplicons remain elusive.

It has now become evident that not all genes within an amplicon are overexpressed when amplified. For example, in the *HER2 *amplicon, only 7 of the 13 genes that map to the smallest region of amplification are expressed at significantly higher levels when amplified [20-22]. Conversely, evidence now suggests that an amplicon may harbour more than one driver [10,11,17,18,23,24]. For instance, within the 8p11.2 amplicon, the expression of FGFR1, PPAPDC1B, WHSC1L1, LSM1 and ZNF103 has been shown to be selectively required for the survival of cancer cells harbouring the amplification of these genes [10,11,17-19,25].

Amplification of the 19q12 locus has been reported to be found in up to 15% of ER-negative breast cancers [9,26]. This amplicon often encompasses the cell cycle regulatory gene *CCNE1*, which has been shown to be overexpressed in a subgroup of ER-negative cancers. Although mRNA and protein expression are more prevalent than gene amplification, *CCNE1 *has been postulated as the driver gene of this amplicon [9,26-28]. There is evidence, however, that genes within this amplicon other than *CCNE1 *are consistently overexpressed when amplified [29], including *POP4 *and *C19ORF2*.

The aims of this study were (i) to characterise the 19q12 amplicon in breast cancer, (ii) to determine the genes that are overexpressed when amplified in this amplicon, (iii) to investigate which of the genes mapping to this amplicon are selectively required for the survival of cells harbouring their amplification, and (iv) to determine if cancer cells with *CCNE1 *gene amplification are dependent on CCNE1 cell cycle-related functions for their survival.

## Material and methods

### Tumour samples

Fresh/frozen primary invasive breast carcinomas from 313 patients were retrieved from independent consecutive cohorts after approval by local Research Ethic Committees from the authors' institutions. None of the patients received pre-operative chemotherapy and/or endocrine therapy. Tumours were either micro-dissected to ensure a tumour cell content > 75% as previously described [9], or one representative section of each tumour was stained with haematoxylin & eosin and only samples with > 70% of neoplastic cells were included, as previously described [19]. A description of the cohort analysed here is presented in Additional file 1 Table S1. Out of the samples included in this study, the aCGH profiles of 95, 24 and 8 cases were previously reported in Natrajan *et al. *[9], Turner *et al. *[19], and Hungermann *et al. *[30], respectively. The remaining samples comprise a series of 31 consecutive HER2-positive breast cancers retrieved from the tissue bank of The Netherlands Cancer Institute (NKI), and a collection of 119 consecutive breast cancers from the tissue bank of the NKI. RNA was available for 48 cases and the gene expression profiles were reported in Natrajan *et al. *[29]. RNA of sufficient quantity and quality was available for 48 cases and the gene expression profiles were reported in Natrajan *et al. *[29]. Immunohistochemistry for ER, progesterone receptor (PR) and HER2 was performed as previously described [9,31]. Histological grade was determined by two pathologists (FCG and JSR-F) using the modified Scarff-Bloom-Richardson system [32]. ER and PR status was determined by immunohistochemical analysis using the 6F11 (1:150) and PgR636 (1:200) antibodies, respectively, as previously described [33]. The Allred scoring system was employed and tumours were considered positive when the score was ≥ 3. *HER2 *gene amplification was defined based on chromogenic *in situ *hybridisation analysis using a US Food and Drug Administrator approved probe (SpotLight *HER2 *amplification probe, Invitrogen, Carlsbad, CA, USA) and/or by inspection of the results of aCGH analysis, as previously described [20]. Tumours were classified into ER-positive/HER2-negative, ER-negative/HER2-negative and HER2-positive subgroups, given i) the results of recent studies demonstrating that the transcriptomic profiles of ER-positive/HER2-negative, ER-negative/HER2-negative and HER2-positive tumours are fundamentally different [34,35], ii) the technical issues related to the assignment of tumour profiled with different platforms into the 'intrinsic' molecular subtypes [36,37], and iii) that these subgroups are currently employed to define the systemic therapy for patients with breast cancer.

### RNA and DNA extraction

DNA and RNA were extracted as previously described [29]. DNA concentration was measured with Picogreen^® ^(Invitrogen, Paisley, UK) according to the manufacturer's instructions, and RNA was quantified using the Agilent 2100 Bioanalyser with RNA Nano LabChip Kits (Agilent Biosystems, Stockport, UK).

### Cell lines

Fifty-six breast cancer cell lines were obtained from ATCC (LGC Standards, Teddington, UK) unless otherwise specified (Additional file 2 Table S2), maintained as previously described [16,19,38] and subjected to aCGH analysis as described below. Out of these cell lines, MDA-MB-157, HCC1569, MDA-MB-231, Hs578T, MCF7, ZR75.1, BT474 and JIMT1 were used as models for the functional characterisation of the 19q12 amplicon.

### Microarray-based comparative genomic hybridisation (aCGH)

The 32K bacterial artificial chromosome (BAC) re-array collection (CHORI) tiling path aCGH platform was constructed at the Breakthrough Breast Cancer Research Centre, as described previously [33,39]. This type of BAC array platform has been shown to be as robust as, and to have comparable resolution with, high density oligonucleotide arrays [40-42]. DNA labelling, array hybridisations and image acquisition were performed as previously described [9]. aCGH data were pre-processed and analysed using an in-house R script (BACE.R) in R version 2.13.0, as previously described [29,33]. In brief, raw Log_2 _ratios of intensity between samples and pooled female genomic DNA were read without background subtraction and normalised in the LIMMA package in R using PrinTipLoess. Outliers were removed based upon their deviation from neighbouring genomic probes using an estimation of the genome-wide median absolute deviation of all probes. After filtering polymorphic BACs a final dataset of 31,367 clones with unambiguous mapping information according to the February 2009 build (hg19) of the human genome [43]. Log_2 _ratios were rescaled using the genome-wide median absolute deviation in each sample, and then smoothed using circular binary segmentation in the DNACopy package as previously described [29,33]. Loss was defined as a circular binary segmentation (cbs)-smoothed Log_2 _ratio < -0.08. Low-level gain was defined as a cbs-smoothed Log_2 _ratio in the range 0.08 to 0.45, corresponding to approximately three to five copies of the locus, whilst gene amplification was defined as having a Log_2 _ratio > 0.45, corresponding to more than five copies [9,33,39]. Threshold values were determined as previously described [9] and validated empirically by means of *in situ *hybridisation methods in previous publications [9,33,39,44-46]. A categorical analysis was applied to the BACs after classifying them as representing amplification (>0.45), gain (>0.08 and ≤ 0.045), loss (< -0.08), or no-change according to their cbs-smoothed Log_2 _ratio values [29,33]. Threshold values were determined and validated as previously described [9]. aCGH data, the analysis history, script and code are publically available online at [47].

### FISH validation of aCGH results

Fluorescence *in situ *hybridisation (FISH) analysis for *19q12 *amplification was carried out as previously described [9,48]. Briefly, a FISH probe mapping to 30.10 to 30.25 Mb on chromosome 19 was generated using the BAC RP11-372I05 and biotin labelled as previously described [48]. Pre-treatment and hybridisation were carried out as previously described [9,48]. Cases were considered amplified if > 50% of neoplastic cells harboured large signal clusters or > 5 signals/nucleus. FISH performed with observers blinded to the results of aCGH analysis (Additional file 3 Figure S1).

### TP53 mutation analysis

The entire coding sequence of the *TP53 *gene (exons 2 to 11) was sequenced as previously described [39,49]. PCR amplification and Sanger sequencing was carried out in the 16 cases with 19q12 amplification using 5 ng of tumour DNA [50] using the DNA Sequencing Kit BigDye Terminator v 3.1 Cycle Sequencing Ready Reaction Mix (Applied Biosystems, Warrington, UK), as described [39]. Sequences were analysed with Mutation Surveyor software (Softgenetics, State College, PA, USA).

### Gene expression analysis

Gene expression profiling of breast cancer samples in the 'Natrajan' dataset was performed using the Illumina human WG6 version 2 expression array as previously described [29]. Raw gene expression values were robust-spline normalised using the Bioconductor lumi package in R (R Foundation, Vienna, Austria) Genes were mapped to their genomic location using the lumiHumanAllv2 annotation database available from Bioconductor. Only Illumina transcript probes with detection *P*-values < 0.01 in > 25% of samples were included; this resulted in a dataset of 12,699 transcriptionally regulated probes with accurate and unequivocal mapping information. Gene-expression data are publicly available at ArrayExpress (accession number: E-TABM-543 [51]. The final dataset comprises 48 cases, whose clinicopathological characteristics are fully described elsewhere [29].

### Identification of genes whose expression correlates with copy number changes

To identify genes whose expression levels correlate with copy number changes, cbs-smoothed Log_2 _ratios from aCGH data were used to assign the aCGH states for each of the genes in the gene expression dataset using the median values for all BACs that overlap with the genomic position of each gene. This resulted in a 1:1 matrix of expression values and aCGH cbs values, which were used for downstream statistical analysis. To define genes that were overexpressed when amplified, a Mann-Whitney U test was performed using categorical aCGH states (that is, amplification versus no amplification) as the grouping variable and the expression of genes as the dependent variable, as previously described [16,29]. *P*-values < 0.05 were considered significant.

### Short interfering RNA (siRNA)-mediated silencing

Nine genes that mapped to the smallest region of amplification on 19q12 were selected for functional evaluation, namely *CCNE1*, *UQCRFS1*, *POP4*, *PLEKHF1*, *C19ORF2*, *C19ORF12*, *VSTM2B*, *ZNF536*, *TSHZ3*. Each gene was targeted with four distinct siRNAs (siGENOME SMARTpool) obtained from Thermo Scientific (Epsom, UK): siCCNE1 - pool M-003213-02; siUQCRFS1 - pool M-020100-01; siPOP4 - pool M-020046-00; siPLEKHF1 - pool M-018423-01; siC19ORF2 - pool M-017399-01; siC19ORF12 - pool M-014731-01; siVSTM2B - pool M-023625-01; siZNF536 - pool M-020506-01; siTSHZ3 - pool M-014119-01; and siCDK2 - pool M-003236-04. siGENOME Non-Targeting siRNA Pool #1 and #2 (siCON, D-001206-13 and D-001206-14) were used as controls. As a positive control (that is, a gene whose silencing is lethal) we employed siRNA pools for PLK1 (M-003290-01) as previously described [44]. To identify conditions that maximise transfection efficiency and minimise lipid-mediated toxic effects, multiple transfection conditions for each cell line were tested. Lipofectamine RNAiMax (Invitrogen, Paisley, UK) and Dharmafect 4 (Thermo Scientific, Epsom, UK) were identified as the most efficient and least toxic for the cell lines studied. Breast cancer cells were transfected with target and control siRNAs (50 nmol/L per well in 100 μL total volume) in 96-well plates, according to the manufacturers' instructions as previously described [44,52]. A total of 1,000 to 5,000 cells were seeded per well that yielded 80 to 90% confluency in the controls at 10 days. Media were replaced every two days and cell viability was assessed using the CellTiter-Glo^® ^assay (Promega, Southampton, UK) as previously described [44]. The average cell survival fraction for each siRNA was calculated relative to that of cells transfected with non-coding control siRNA.

### Drug sensitivity assays

For assessment of drug sensitivity, cell lines were plated and transfected with target or control siRNAs in 96-well plates. At 48 hours post transfection, media were supplemented with serial dilutions (10^-4^M to 10^-9^M) of doxorubicin, cisplatin, paclitaxel, or the CDK1, CDK2 and CDK9 inhibitor AZD5438 (Tocris Biosciences, Bristol, UK), essentially as previously described [44]. All experiments were performed in triplicate. Survival was assessed with CellTiter-Glo^® ^cell viability assay after seven days of drug treatment and survival fraction compared to siRNA vehicle treated cells. Survival curves and estimated SF_50 _(the drug concentration used following which 50% of cells survive) were calculated using non-linear regression with GraphPad Prism V5.0 (La Jolla, CA, USA).

### Cell cycle analysis

FACS analysis for cell cycle assessment was performed with Propidium Iodide staining five days after transfection as described previously [52].

### Western blotting

Total protein lysates (40 μg) from treated and untreated cells were separated by SDS-PAGE according to standard protocols, and immunoblotting was carried out using primary antibodies directed against CCNE1 (HE-12, Abcam ab3927, Cambridge, UK), CDK2 (2546, Cell Signaling Technology, Danvers, MA, USA), phospho-CDK2 (2561, Cell Signaling), PLEKHF1 (20389-1-AP, Proteintech, Chicago, IL, USA), POP4 (Rpp29, sc-23048, Santa Cruz Biotechnology, Santa Cruz, CA, USA), TSHZ3 (sc-134132, Santa Cruz), and CCNB1 (4135, Cell Signaling), and against β-tubulin as loading control (5346, Cell Signaling). Protein expression levels after siRNA silencing were assessed by western blotting 72 hours after transfection of the cells and protein bands quantified using ImageJ 1.44p software (NIH, Bethesda, MD, USA) [53].

### Real-time quantitative reverse transcriptase PCR (qRT-PCR)

First strand synthesis was performed as previously described [9], and qRT-PCR was performed using TaqMan chemistry on the ABI Prism 7900HT (Applied Biosystems), using the standard curve method. Assays for CCNE1, PLEKHF1, UQCRFS1, C19ORF12, C19ORF2, VSTM2B, ZNF536, TSHZ3, TFRC and MRPL19 were purchased from Applied Biosystems. Expression levels were normalised to those of TFRC and MRPL19 (Assay on demand ID: Hs00174609_m1-TFRC, Hs00608522_g1-MRPL19, Hs01026536_m1-CCNE1, Hs00759096_s1-PLEKHF1, Hs00705563_s1-UQCRFS1, Hs01026936_m1-C19ORF2, Hs00198357_m1-POP4, Hs01107514_m1-C19ORF12, Hs0416833_m1-VSTM2B, Hs00206981_m1-ZNF536, Hs01583885_m1-TSHZ3, Hs01548894_m1-CDK2, Hs99999188_m1-CCNB1). For the assessment of RNA levels after gene silencing, RNA was extracted using Trizol (Invitrogen, Paisley, UK), and transcript levels were assessed 48 hours after transfection. The level of siRNA silencing was measured relative to that of cells transfected with non-coding control siRNA.

### Statistical analysis

All statistical analysis was performed with R version 2.13.0 or GraphPad Prism version 5.0. All statistical tests performed adopted 95% confidence intervals. A two-tailed *P-*value of < 0.05 was considered significant.

## Results

### 19q12 amplification is associated with grade III ER-negative breast cancers

**Figure 1:**
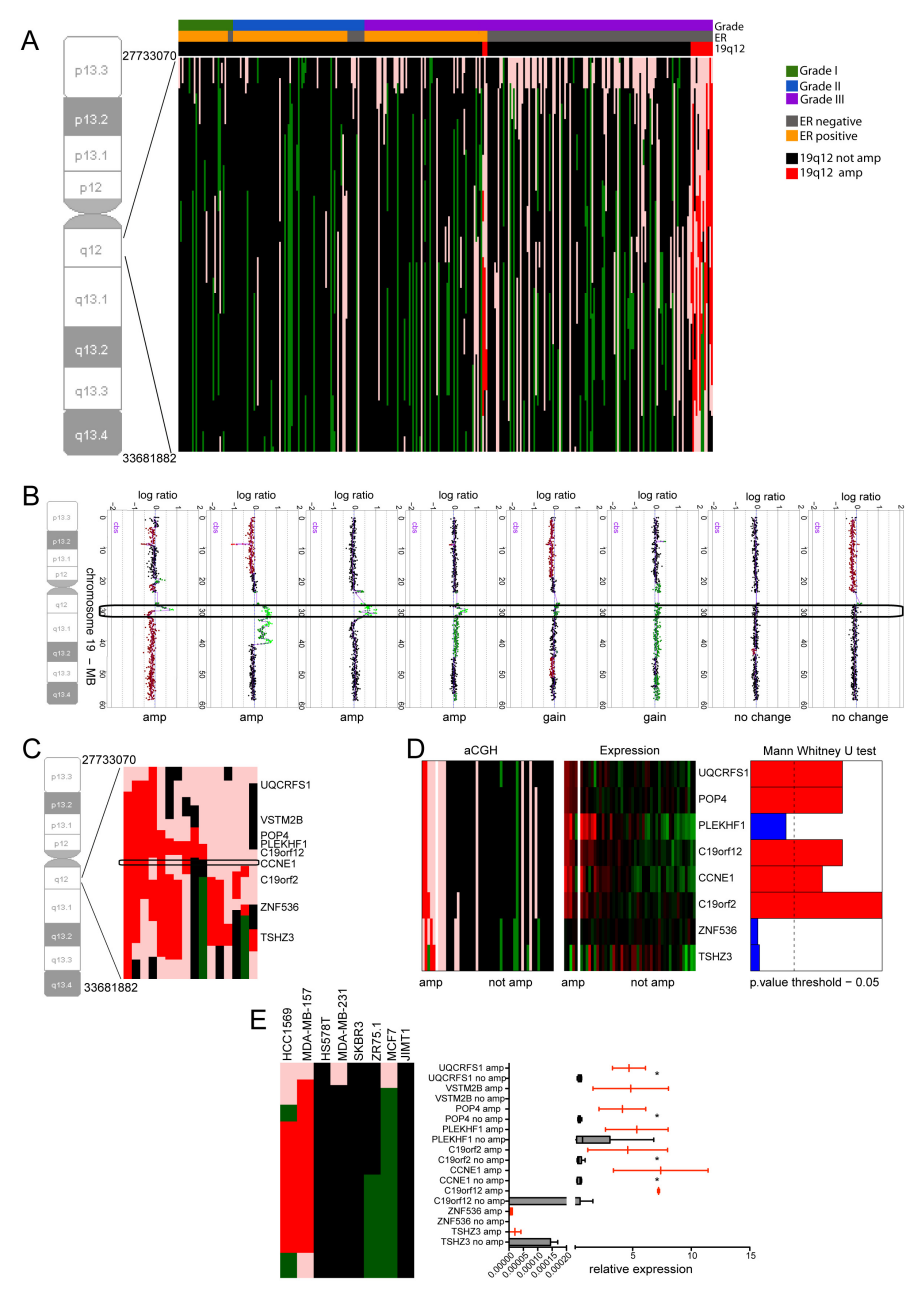
**Patterns of 19q12 amplification and mRNA expression of genes mapping to the 19q12 amplicon**. **(A) **aCGH heatmap of chromosome 19q12 in 313 primary breast cancers. Tumours were ordered according to histological grade, oestrogen receptor (ER) status and 19q amplification status. **(B) **Chromosome 19 ideogram and aCGH plots illustrating the patterns of 19q12 amplification (bright green, amp), copy number gain (green, gain) and no copy number change (black, no change) in primary breast cancers. **(C) **aCGH heatmap of region of amplification in 16 breast cancers harbouring amplification of the 19q12 locus. *CCNE1 *(box) was amplified in 5/16 cancers. **(D) **Heatmaps depicting aCGH states (left) and gene expression values (middle) in 48 primary breast cancers with or without 19q12 amplification. Genes are ordered according to their chromosomal position and tumours grouped according to the 19q12 amplification status. Bar plots (right) depict the Mann-Whitney U test results for the comparison of gene expression as a continuous variable and gene amplification as the grouping variable. In red, genes with *P-*values < 0.05 (dotted line). **(E) **aCGH heatmap of the 19q12 amplicon in breast cancer cells with (MDA-MB-157, HCC1569) or without (MDA-MB-231, Hs578T, MCF7, ZR75.1, BT474, JIMT1) 19q12 amplification. Box-and-whiskers plots showing the expression of genes within the amplified region in cell lines with (red) and without (grey) 19q12 amplification. *: genes significantly overexpressed when amplified (Mann-Whitney U test, *P* < 0.05). In all aCGH heatmaps (A, C, D, E), green: copy number loss, black: no copy number change; pink: copy number gain; bright red: gene amplification. In the microarray gene expression heatmap (D), green: down-regulation; red: up-regulation.

To determine the clinicopathological correlates of the 19q amplification, 313 frozen breast cancers were subjected to aCGH analysis [9]. Amplification at 19q12 (27.8 to 32.9 Mb) was found in 16/313 (5.1%) of all tumours, and 7.8% (16/188) of grade III cancers (Figure 1A-C; Table 1). Amplification of 19q12 was significantly associated with histological grade III (Fisher's exact test *P *= 0.018, Table 1), lack of ER expression (Fisher's exact test, *P *= 0.0042) and ER-negative/HER2-negative subtype (Chi-square test *P *= 0.0056, Table 1). Given the small number of samples in the subgroup analysis of the clinical subtypes, we have compared the prevalence of 19q12 amplification in ER-negative/HER2-negative vs the other subtypes using the Fisher's exact test, which confirmed a significance association between 19q12 amplification and ER-negative/HER2-negative breast cancers (*P *= 0.0024).

**Table 1:** 19q12 amplification in 313 breast cancers and clinicopathological associations

		19q12 Amp n (%)	19q12 Not amp n (%)	*P-*value	*CCNE1* Amp (%)	*CCNE1* Not amp (%)	*P-*value
Whole cohort		16 (5.1%)	297 (94.9%)	NA	5 (1.6%)	308 (98.4%)	NA

Histological grade*				**0.0018^+^**			0.1693^+^

	I/II	0 (0%)	109 (100%)		0 (0%)	109 (100%)	

	III	16 (8%)	188 (92%)		5 (2%)	199 (98%)	

ER				**0.0042^+^**			**0.01984^+^**

	Positive	3 (2%)	166 (98%)		0 (0%)	169 (100%)	

	Negative	13 (9%)	131 (91%)		5 (3.5%)	139 (96.5%)	

HER2				0.5398**^+^**			0.9801**^+^**

	Positive	2 (3%)	62 (97%)		1(1.6%)	63(98.4%)	

	Negative	14 (6%)	235 (94%)		4(1.6%)	245(98.4%)	

Subtypes				0.0056**^			0.0500**^

	ER-/HER2-	11 (11%)	90 (89%)	0.0024***	4 (4%)	97 (96%)	0.0399***

	HER2	2 (3%)	62 (97%)		1 (2%)	63 (98%)	

	ER+/HER2-	3 (2%)	145 (98%)		0 (0%)	148 (100%)	

Amp, amplified; *CCNE1*, cyclin E1; ER, oestrogen receptor; NA, not applicable; Not amp, not amplified.

*Histological grade determined by the modified Scarff-Bloom-Richardson system [32].

Significant *P-*values are highlighted in bold.

+: Fisher's exact test

**: Chi-square test

***: Comparison between ER-/HER2- vs the remaining subtypes by Fisher's exact test.

^: Given that > 20% of the groups have n < 5, the Chi-square *P-*value should be interpreted with caution.

Amplification of the 19q12 locus was shown to be complex and this amplicon displayed multiple core regions recurrently amplified in primary breast cancers (Figure 1C). Within these cores, nine genes were identified, including *CCNE1 *(Table 2). Although *CCNE1 *has been proposed as a driver of this amplicon in breast cancer [14,26], *CCNE1 *was amplified in only 5 out of the 16 primary breast cancers harbouring amplification of the 19q12 locus (Figure 1C).

**Table 2:** Genes mapping to the 19q12 region of amplification

Gene symbol	Gene name	Position (kb)	Copy number and expression Pearson's correlation (r)	Overexpressed when amplified (MWU) *P-*value
UQCRFS1	ubiquinol-cytochrome c reductase, Rieske iron-sulfur polypeptide 1	29,698 to 29,704	0.65*	**0.0018**
VSTM2B	V-set and transmembrane domain containing 2B	30,017 to 30,055	NA	NA
POP4	processing of precursor 4, ribonuclease P/MRP subunit (S. cerevisiae)	30,097 to 30,107	0.77*	**0.0018**
PLEKHF1	pleckstrin homology domain containing, family F (with FYVE domain) member 1	30,156 to 30,166	0.44*	0.0868
C19ORF12	Uncharacterised protein C19ORF12	30,190 to 30,206	0.63*	**0.0018**
CCNE1	Cyclin E1	30,303 to 30,315	0.66*	**0.0071**
C19ORF2	RPB5-mediating protein	30,433 to 30,507	0.81*	**0.0001**
ZNF536	Zinc finger protein 536	30,863 to 31,049	-0.03	0.6064
TSHZ3	Teashirt zinc finger homeobox 3	31,766 to 31,840	0.08	0.5426

Position refers to the genomic location from the Ensembl genome browser based on the February 2009 hg19 build of the genome

*• P *< 0.05; significant *P-*values are highlighted in bold; MWU (Mann-Whitney U test).

### Potential 19q12 amplicon drivers

Genes within an amplicon that are overexpressed when amplified have been shown to constitute potential amplicon drivers and therapeutic targets [9-12,17-19,29,54]. To determine which of the amplified genes in the 19q12 amplicon were significantly overexpressed when amplified, aCGH and microarray expression data from 48 microdissected grade III breast cancers were interrogated [29,36]. This analysis revealed that out of all the genes mapping to the 19q12 amplification with detectable gene expression, *UQCRFS1*, *POP4*, *PLEKHF1*, *C19ORF12*, *CCNE1 *and *C19ORF2 *had expression levels that significantly correlated with gene copy number (Pearson's correlation r value = 0.65, 0.77, 0.44, 0.63, 0.66, 0.81, respectively; all *P* < 0.05) and *UQCRFS1*, *POP4*, *C19ORF12*, *CCNE1 *and *C19ORF2 *were significantly overexpressed when amplified (Mann-Whitney U test *P* < 0.05) (Figure 1D, Table 2). *VSTM2B *expression was undetected in any of the tumours analysed.

To identify models for the study of the genes overexpressed when amplified in the 19q12 amplicon, we first subjected 56 breast cancer cell lines to aCGH analysis and identified two cell lines (that is, HCC1569 and MDA-MB-157), which harbour amplification of 19q12 (minimal amplified region from 29.17 to 32.82 Mb). In a way akin to the majority of primary breast cancers harbouring 19q12 amplification [9], the only cell lines harbouring amplification of this locus were ER-negative/HER2-negative (MDA-MB-157) or ER-negative/HER2-positive (HCC1569) [16,38]. A panel of phenotypically matched (in terms of ER, PR and HER2 expression) breast cancer cell lines were selected and used as controls (that is, MDA-MB-231, Hs578T, MCF7, ZR75.1, JIMT1 and BT474; Additional file 2 Table S2). Quantitative RT-PCR of cDNA from cancer cells harbouring 19q12 amplification or lacking amplification of this locus revealed that *UQCRFS1*, *POP4*, *CCNE1 *and *C19ORF2 *genes were overexpressed when amplified (*P* < 0.05, Mann Whitney U test, Figure 1E), whereas *VSTM2B*, *ZNF536 *and *TSHZ3 *expression could not be detected in the majority of cell lines at the mRNA level.

Taken together, our results suggest that *UQCRFS1*, *POP4*, *C19ORF12*, *CCNE1 *and *C19ORF2 *are overexpressed in primary breast cancers and/or breast cancer cell lines harbouring their amplification, and may constitute potential drivers of this amplicon.

### CCNE1, POP4, PLEKHF1 and TSHZ3 are selectively required in cells harbouring their amplification

**Figure 2:**
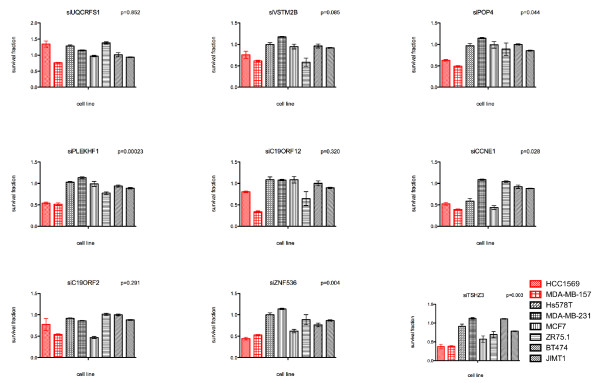
**Impact of RNA interference silencing of genes mapping to the 19q12 amplicon on cell viability**. Bar plots of the survival fraction relative to non-targeting siRNA control of breast cancer cells with (red) or without (black) 19q12 amplification upon transfection with short interfering RNA (siRNA) SMARTpools targeting *UQCRFS1*, *VSTM2B*, *POP4*, *PLEKHF1*, *C19ORF12*, *CCNE1*, *C19ORF2*, *ZNF536 *and *TSHZ3*. Cell viability was assessed after 10 days with CellTiter-Glo^® ^cell viability assay. *P-*values of t-test of the survival fraction between cancer cells harbouring the amplification of the gene tested and those lacking its amplification (*P* < 0.05 was considered significant). Error bars represent the standard error of the mean of three replicates.

**Figure 3:**
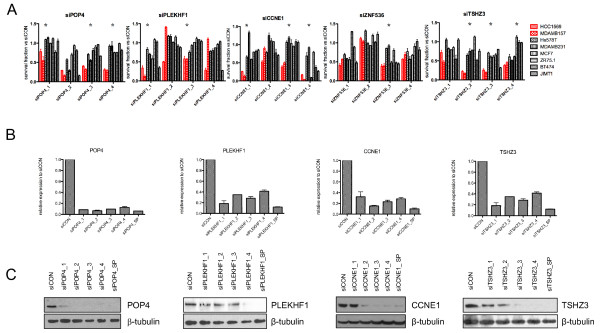
***PLEKHF1*, *POP4 *and *TSHZ3 *expression is selectively required for the survival of 19q12-amplified breast cancer cells**. **(A) **Barplots illustrating the survival fractions of cancer cells with (red) or without (black) 19q12 amplification upon transfection with short interfering RNA (siRNA) targeting *POP4*, *PLEKHF1*, *UQCRFS1*, *CCNE1, ZNF536*, and *TSHZ3 *relative to siRNA controls (siCON) of individual duplex oligonucleotides (1 to 4). Error bars represent the standard error of the mean of three replicates. Reduced cell viability effects were considered validated if at least two individual oligonucleotides caused a significantly higher loss of viability of cancer cells harbouring 19q12 amplification than in those lacking amplification of this locus (* depicts significant t-test *P-*values for the comparison of the survival fraction between cells with and without amplification of the target gene for an individual oligonucleotide). siZNF536 failed to validate with two or more individual siRNA oligonucleotides. **(B) **Effects on target mRNA expression after silencing of *PLEKHF1*, *POP4*, *TSHZ3 *and *CCNE1 *with individual oligonucelotides in MDA-MB-157 cells as determined by quantitative RT-PCR. **(C) **Effects on target protein expression after silencing of *PLEKHF1*, *POP4*, *TSHZ3 *and *CCNE1 *with Dharmacon individual oligonucelotides and smartpool (SP) in MDA-MB-157 (CCNE1) and MCF7 cells (PLEKHF1, POP4, and TSHZ3) as determined by Western blotting.

There are several lines of evidence to suggest that one of the characteristics of amplicon drivers is that their expression is selectively required for the survival of cancer cells harbouring their amplification [9-12,19]. Given the complexity of amplification at this locus, we chose to assess the potential amplicon drivers by siRNA silencing of all genes mapping to the 19q12 amplicon (that is, *UQCRFS1*, *VSTM2B*, *POP4*, *C19ORF12*, *PLEKHF1*, *CCNE1*, *C19ORF2, ZNF536 *and *TSHZ3*) regardless of their expression levels. Silencing of *CCNE1*, *PLEKHF1*, *POP4*, *ZNF536 *and *TSHZ3 *expression selectively reduced cell viability in cancer cells harbouring 19q12 gene amplification but had a significantly lower impact on the survival of breast cancer cells lacking amplification of this locus (t-test *P* < 0.05; Figure 2). Upon siRNA pool deconvolution, the observation that silencing of *CCNE1*, *PLEKHF1*, *POP4 *and *TSHZ3 *is selectively lethal in cancer cells harbouring their amplification was validated (that is, two or more individual siRNAs were selectively lethal in cancer cells harbouring amplification of the gene silenced; Figure 3A). siRNA silencing of these genes was confirmed at the mRNA and protein levels (Figures 3B, C).

**Figure 4:**
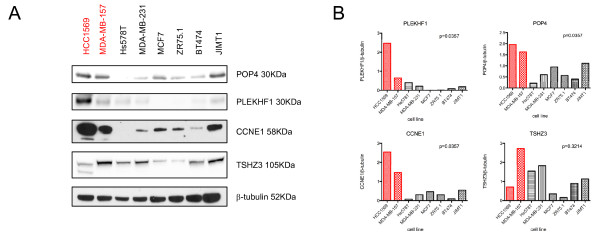
**PLEKHF1, POP4, and CCNE1 are overexpressed when amplified at the protein level**. **(A) **Western blot analysis of cell line panel with antibodies against POP4, PLEKHF1, CCNE1 and TSHZ3 in the breast cancer cell lines studied. In red, breast cancer cell lines harbouring amplification of the 19q12 locus. **(B) **Bar plots depicting POP4, PLEKHF1, CCNE1 and TSHZ3 protein expression quantified relative to the β-tubulin loading control, using ImageJ 1.44p (NIH, USA).

Assessment of the protein levels of POP4, PLEKHF1, CCNE1 and TSHZ3 was performed in the cell lines used in this study. Expression levels of POP4, PLEKHF1 and CCNE1 were significantly higher in cancer cells harbouring amplification of the genes encoding these proteins than in cancer cells devoid of amplification of these loci (Figure 4, Mann-Whitney U test *P *< 0.05). No significant association between *TSHZ3 *gene amplification and protein expression was observed, even when corrected for proliferation levels, as defined by CCNB1 expression levels (data not shown).

**Figure 5:**
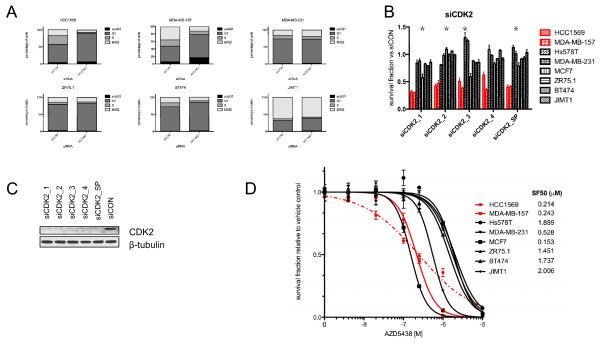
**Cancer cells harbouring *CCNE1 *gene amplification show increased sensitivity to CDK2 silencing and chemical inhibition**. **(A) **The fraction of cells in each phase of the cell cycle are shown following transfection with CCNE1 short interfering RNA (siRNA) and control (siCON) in cell lines with (HCC1569 and MDA-MB-157) and without (MDA-MB-231, ZR75.1, BT474 and JIMT1) *CCNE1 *amplification. **(B) **Bar plots illustrating the survival fraction of cells following transfection with CDK2 siRNA using individual duplex oligonucelotides (1 to 4) and a SMARTpool (SP) in cancer cells harbouring (red) or lacking (black) 19q12 amplification. Error bars represent the standard error of the mean of three replicates. Survival fractions are shown relative to those of siCON transfected cells. (* depicts significant t-test *P *values for the comparison of the survival fraction between cells with and without amplification of the target gene for an individual oligonucleotide). **(C) **Effects on CDK2 protein expression of *CDK2 *siRNA silencing with Dharmacon individual oligonucelotides and SMARTpool (SP) in MDA-MB-157 cells. **(D) **Dose response curves of breast cancer cell lines with (red) or without (black) 19q12 amplification to the CDK1, CDK2, and CDK9 inhibitor ADZ5438.

To test whether silencing of these genes affects cell cycle in cancer cells with or without 19q12 amplification, DNA content was measured upon gene silencing. Upon *PLEKHF1*, *POP4 *or *TSHZ3 *siRNA-mediated gene silencing, slight but not statistically significant increases in the subG1 fraction and decreases in the G2/M fraction were observed in cancer cells with (that is, MDA-MB-157 and HCC1569) or without (that is, MDA-MB-231, JIMT1, ZR75.1 and BT474) 19q12 amplification (Additional file 4 Figure S2). These observations suggest that the silencing of these genes does not significantly alter the cell cycle profile. On the other hand, in cancer cells harbouring 19q12 amplification, *CCNE1 *silencing resulted in a significant decrease in the fraction of cells in S/G2 and increase in the proportion of cells in G1 and subG1 cell cycle phases (*P* < 0.0001, Chi-square test, Figure 5A).

To assess whether amplification and overexpression of *CCNE1*, *PLEKHF1*, *POP4 *and *TSHZ3 *would alter the response of breast cancer cells to common chemotherapeutic agents, we subjected cancer cells with or without 19q12 gene amplification to treatment with doxorubicin, cisplatin and paclitaxel upon silencing of these genes. No significant difference in the sensitivity of these cells to the chemotherapy agents tested was observed upon silencing of *PLEKHF1*, *POP4 *or *TSHZ3 *compared to non-targeting siRNAs (data not shown). *CCNE1 *siRNA-mediated silencing, however, was found to reduce the sensitivity of cancer cells to doxorubicin, cisplatin and paclitaxel in cancer cells harbouring 19q12 amplification cells but not in cancer cells lacking amplification of this locus (Additional file 5 Figure S3). This is not surprising, given that these chemotherapy agents target cycling cells and, here, we demonstrate that *CCNE1 *siRNA-mediated silencing leads to G1 arrest.

### CDK2 silencing and chemical inhibition is selectively lethal in cancer cells harbouring CCNE1 gene amplification

Given that i) cancer cells harbouring *CCNE1 *gene amplification depend on CCNE1 expression for their survival, ii) that one of the main functions of CCNE1 is to activate CDK2 in the transition from G1/S phase [55], and iii) that *CCNE1* RNAi-mediated silencing led to G1 arrest, we posited that cancer cells harbouring *CCNE1 *gene amplification would be dependent on CDK2 expression and kinase activity for their survival. siRNA-mediated *CDK2 *silencing resulted in a significantly higher reduction in cell survival of cancer cells harbouring *CCNE1 *gene amplification than in cancer cells devoid of amplification of this gene (t-test, *P* < 0.05, Figures 5B, C). Furthermore, cancer cells harbouring *CCNE1 *gene amplification displayed a higher sensitivity to the CDK1, CDK2 and CDK9 small molecule inhibitor AZD5438 [56,57] than cancer cells lacking *CCNE1 *gene amplification (t-test, *P *= 0.019, Figure 5D). Moreover, primary breast cancers harbouring *CCNE1 *amplification were found to have higher levels of CDK2 mRNA expression, adjusted for proliferation using the mRNA levels of CCNB1 [58], than breast cancers devoid of *CCNE1 *amplification (*P *= 0.02789, t-test, data not shown).

Taken together, these data provide evidence that tumours harbouring 19q12 amplification have more than one 'driver' and suggest that cancer cells with *CCNE1 *amplification depend on CDK2 expression and kinase activity for their survival.

## Discussion

Here we demonstrate that the 19q12 locus is amplified in 5.1% of all invasive breast cancers and that this amplification is preferentially found in grade III ER-negative breast cancers. This is consistent with previous observations that suggested that 19q12 amplification is found in 5 to 15% of breast cancers and is associated with ER-negative disease [9,26,27,29]. Furthermore, through a combination of genomic profiling of primary breast cancers and breast cancer cell lines and RNAi experiments, we have demonstrated that the 19q12 amplicon may contain more than one 'driver'.

Previous studies have suggested that *CCNE1 *is the likeliest driver of the 19q12 amplicon [26,27]. Although our results support the contention that *CCNE1 *is one of the drivers of this amplicon, in this study *CCNE1 *was amplified only in 5 out of 16 breast cancers harbouring 19q12 amplification. These observations are in agreement with those from studies of the 19q12 amplicon in gastric cancer, where *CCNE1 *was amplified only in a subset of cases harbouring 19q12 amplification [59], and with those of previous studies that have found a prevalence of *CCNE1 *amplification in 1.2% to 1.4% of primary breast cancers [14,60]. Taken together, these lines of evidence suggest that *CCNE1 *may not be the sole driver of this amplicon and that other genes within the 19q12 amplicon may also constitute drivers. Consistent with this hypothesis, we observed that cancer cells harbouring 19q12 amplification require the expression of not only CCNE1, but also PLEKHF1, POP4 and TSHZ3 for their survival.

*PLEKHF1 *(pleckstrin homology domain containing, family F (with FYVE domain) member 1) encodes a protein that is known to induce apoptosis through the lysosomal-mitochondrial pathway and triggers caspase-independent apoptosis [61]. Recent evidence suggests that this process involves the recruitment of phosphorylated p53 and that silencing of endogenous p53 impairs its function [62]. Despite its reported role in apoptosis, PLEKHF1 siRNA-mediated silencing in cancer cells harbouring its amplification did not lead to an increase in the sub-G0 proportion of cells. It could be speculated that in a *TP53 *mutant background (as was the case of HCC1569 and MDA-MB-157; Additional file 2 Table S2), *PLEKHF1 *gene amplification and overexpression may confer a selective advantage through mechanisms other than through the recruitment of phosphorylated p53. Regrettably, no breast cancer cell lines harbouring *PLEKHF1 *gene amplification and wild-type *TP53 *were available to test this hypothesis. All primary breast cancers with *PLEKHF1 *amplification, however, were found to harbour *TP53 *mutations, suggesting that *PLEKHF1 *amplification and *TP53 *gene mutations may have epistatic interactions and that *TP53 *mutational status should be taken into account in the evaluation of the potential role of PLEKHF1 as a therapeutic target. *POP4 *(processing of precursor 4, ribonuclease P/MRP subunit (S. cerevisiae)) encodes one of the protein subunits of the small nucleolar ribonucleoprotein complexes and is involved in the processing of precursor RNAs. Further studies investigating the mechanisms that lead to a survival advantage in cancer cells harbouring amplification and overexpression of *PLEKHF1 *and *POP4 *are warranted. *TSHZ3 *(teashirt zinc finger homeobox 3) is a zinc finger transcription factor and has been shown to be involved in muscle cell differentiation [63,64]. Given that the *TSHZ3 *gene promoter is reported to be frequently methylated in primary breast cancers and breast cancer cell lines [65], this gene has been suggested to display tumour suppressor function. Our results do not corroborate this hypothesis and suggest that *THSZ3 *is one of the drivers of the 19q12 amplicon, given that we demonstrate here that *THSZ3 *is amplified in 2.6% of breast cancers, and that its silencing is selectively lethal in cancer cells harbouring its amplification. Although protein expression is not directly correlated with amplification, the breast cancer cell lines harbouring 19q12 amplification displayed either the highest levels of THSZ3 protein expression (that is, MDA-MB-157) or the presence of a THSZ3 isoform (HCC1569, Figure 4).

Although *CCNE1 *amplification was restricted to a subset of cancers harbouring 19q12 amplification, we set out to investigate if breast cancer cells harbouring *CCNE1 *amplification would be selectively dependent on the expression of this gene for their survival. siRNA-mediated silencing of *CCNE1 *had a significantly greater effect on the survival of cancer cells harbouring *CCNE1 *amplification than in those lacking its amplification. This is in agreement with a recent study in ovarian cancer that demonstrated that reduction of CCNE1 expression significantly inhibited cell growth in CCNE1 expressing cells, with a more profound effect in ovarian cancer cells harbouring *CCNE1 *gene amplification [66]. Moreover, forced expression of CCNE1 in cells with low expression has been previously shown to result in increased cell proliferation [66]. Here we demonstrate that *CCNE1* siRNA silencing in breast cancer cells harbouring *CCNE1 *gene amplification, but not in those lacking this amplification, resulted in a significant arrest in G1 (Figure 5 and Additional file 4 Figure S2). These findings provide a rationale for the apparent selective reduction in sensitivity to chemotherapy agents caused by *CCNE1 *siRNA silencing reported in ovarian cancer cells harbouring *CCNE1 *gene amplification [67], and described here in breast cancer cells harbouring *CCNE1 *gene amplification (Additional file 5 Figure S3).

Progression through the G1/S phase of the cell cycle is regulated through the partnership of CDK2 with its regulatory subunit CCNE1 [55]. Given that *CCNE1 *silencing in cancer cells harbouring *CCNE1 *gene amplification leads to G1 arrest, we tested whether these cells would be dependent on CDK2 expression and kinase activity for their survival. *CDK2 *siRNA silencing and inhibition of CDK2 kinase activity using a CDK1, CDK2 and CDK9 inhibitor (AZD5438) resulted in significantly higher reduction in survival of cancer cells harbouring *CCNE1 *gene amplification. This is in agreement with a recent study based on the analysis of the conditional mouse models MMTV-Low Molecular Weight (LMW)-*Ccne1*; *Tp53*^+/-^; *Cdk2*^+/+^, MMTV-LMW-*Ccne1*; *Tp53*^+/-^; *Cdk2*^+/- ^and MMTV-LMW-*Ccne1*; *Tp53*^+/-^; *Cdk2*^-/-^. While mice with at least one functional copy of *Cdk2 *consistently developed mammary gland tumours, *Cdk2*^-/- ^mice did not develop tumours through 24 months. Furthermore, administration of two Cdk inhibitors delayed the progression of mammary gland tumours in MMTV-LMW-*Ccne1*; *Tp53*^+/-^; *Cdk2*^+/+ ^mice [68]. It should be noted that although MCF7 cells showed a similar sensitivity to the CDK1, CDK2 and CKD9 inhibitor AZD5438, *CDK2 *siRNA silencing had a significantly more limited impact on the viability of these cells, suggesting that the sensitivity of MCF7 cells to AZD5438 is unlikely to be caused by inhibition of CDK2. Taken together, these results demonstrate that breast cancer cells harbouring *CCNE1 *gene amplification are dependent on CDK2 expression and kinase activity for their survival and suggest that *CCNE1 *amplification may constitute a potential biomarker of sensitivity to CDK2 inhibitors. It should be noted, however, that CDK1, CDK2 and CDK9 inhibitors may also be efficacious in a subgroup of ER-positive breast cancers, given that MCF7 cells also show sensitivity to AZD5438. Analysis of the results of clinical trials testing CDK inhibitors in breast cancer patients (for example, "Maximum Tolerated Dose (MTD) of Liposomal Doxorubicin in Combination With Seliciclib for Patients With Metastatic Triple Negative Breast Cancer" trial; http://clinicaltrial.gov identifier NCT01333423) are warranted.

## Conclusion

Here we have demonstrated that *CCNE1 *is a driver of the 19q12 amplicon and that cancer cells harbouring *CCNE1 *gene amplification display an increased sensitivity to *CDK2 *RNAi-mediated silencing and chemical inhibition. It should be noted, however, that *CCNE1 *was shown to be amplified only in a subset of breast cancers harbouring 19q12 amplification. siRNA silencing of all genes mapping to the 19q12 amplicon revealed the existence of genes other than *CCNE1 *whose expression is selectively required for the survival of cancer cells harbouring amplification of this locus. Our results suggest that drivers other than *CCNE1 *may exist in the 19q12 amplicon.

## Abbreviations

aCGH: microarray comparative genomic hybridisation; BAC: Bacterial Artificial Chromosome; CDK2: cyclin-dependent kinase 2; CHORI: Children's Hospital Oakland Research Institute; BACE: Breakthrough Array CGH Expression; cbs: circular binary segmentation; ER: oestrogen receptor; FACS: fluorescence-activated cell sorting; FISH: fluorescence *in situ *hybridisation; MTD: Maximum Tolerated Dose; NKI: Netherlands Cancer Institute; PR: progesterone receptor; Mb: megabase(s); qRT-PCR: Real-time quantitative reverse transcriptase PCR; siRNA: small interfering RNA.

## Competing interests

The authors declare that they have no competing interests.

## Authors' contributions

RN and JRF conceived the study. RN, DW, MBK, MA, KK, FCG and AL carried out the experiments. RN and AM analysed the data. HH, MVV, BK, FR and JP provided samples. RN, PW, BW and JRF wrote the manuscript. All authors read and edited the manuscript, and approved its final version.

## Supplementary Material

Additional file 1 **Table S1**. Phenotypic characteristics of primary breast tumours analysed by microarray-based comparative genomic hybridisation. aCGH results previously reported, Natrajan *et al. *[9]., Hunngermann *et al. *[30], and Turner *et al. *[19]. Histological grade assessed by the modified Scarff-Bloom-Richardson system [32]. Click here for file

Additional file 2 **Table S2**. Details of the 56 breast cancer cell lines subjected to microarray-based comparative genomic hybridisation. ER status, *HER2 *gene amplification status and p53 protein levels and mutational status (adapted from Neve *et. al. *[38], Arriola *et al. *[20] and [69] *p53 mRNA levels derived from Mackay *et al. *[16]. p53 protein levels and mutational status (obtained from COSMIC [70] and Neve *et al. *[38]). "Positive", detectable protein expression; MUT, mutant; "Negative", no detectable protein expression; WT, wild-type. Click here for file

Additional file 3 **Figure S1. Correlation of aCGH smoothed Log_2_ratios and FISH**. Microarray-CGH chromosome 19 plots illustrating the patterns of 19q12 amplification in two primary breast cancers (bright green, circular binary segmentation (cbs)-smoothed Log_2 _ratio > 0.45), copy number gain in two primary breast cancers (green, circular binary segmentation (cbs)-smoothed Log_2 _ratio > 0.08 to ≤ 0.45 and no copy number change in two primary breast cancers (black, circular binary segmentation (cbs)-smoothed Log_2 _ratio > -0.08 to < 0.08). Log_2 _ratios (x-axis) are plotted against BAC position (y-axis). Red: loss, green: gain, bright green: amplification, purple line: smoothed cbs ratios; amp: 19q12 amplified, gain: 19q12 copy number gain, no change: 19q12 no copy number change. Fluorescence *in situ *hybridisation (FISH) confirmation of 19q12 copy number status with a biotin-labelled BAC probe (RP11-327I05, mapping to 30.10 to 30.25 Mb, red). Top two cases show amplification with > 5 copies per nucleus, middle two cases low level gain with three to five copies per nucleus, and bottom two cases no copy number change with two copies per nucleus. Click here for file

Additional file 4 **Figure S2**. Effect of RNA interference gene silencing on cell cycle profiles. Cell cycle profiles of cancer cells harbouring 19q12 amplification (that is, MDA-MB-157 and HCC1569) or lacking amplification of this locus (MDA-MB-231, JIMT1, ZR75.1 and BT474) after RNAi silencing of *POP4*, *PLEKHF1 *and *TSHZ3*. Note the slight increase in the fraction of subG1 in amplified cells, but no difference in the proportion of G1 or S/G2 between cell lines with or without amplification at 19q12. Click here for file

Additional file 5 **Figure S3**. *CCNE1 *gene silencing confers resistance to conventional chemotherapy agents. **(A) **Dose response curves following treatment with paclitaxel after *CCNE1 *silencing using short hairpin RNAs in cancer cells harbouring *CCNE1 *gene amplification (that is, MDA-MB-157 and HCC1569) or lacking amplification at this locus (that is, MDA-MB-231 and ZR75.1). **(B) **Dose response curves after *CCNE1 *silencing to i) doxorubicin, ii) cisplatin and iii) paclitaxel in cancer cells harbouring (MDA-MB-157) or lacking (ZR75.1) 19q12 amplification. Click here for file
