# Supplementary material for: Functional characterization of the 19q12 amplicon in grade III breast cancers
Source: Breast Cancer Res. 2012 Mar 20;14(2):R53. doi: 10.1186/bcr3154 (PMC3446387; doi:10.1186/bcr3154)

Supplementary Figure 1

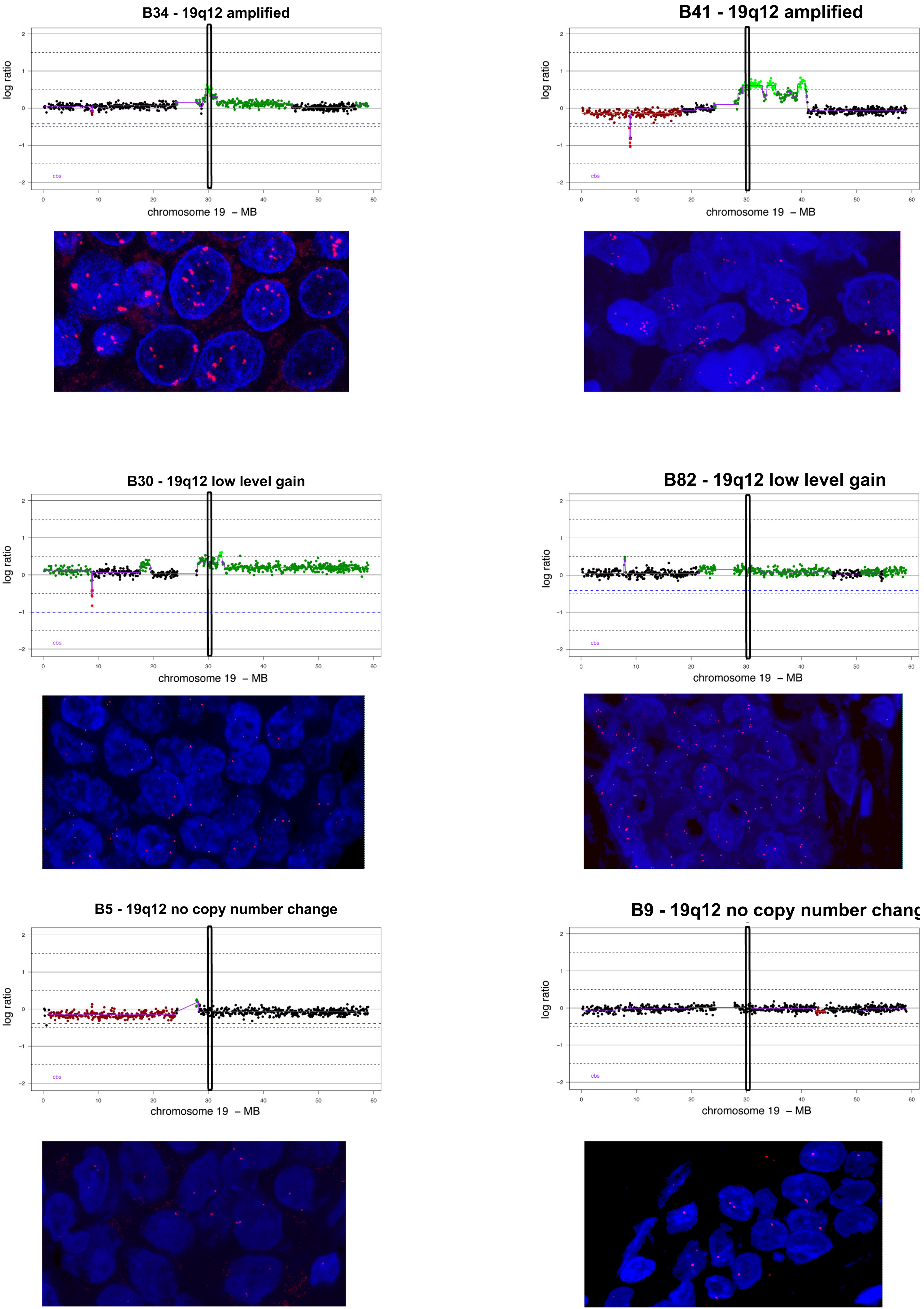

Supplement: Additional file 1 — Table S1. Phenotypic characteristics of primary breast tumours analysed by microarray-based comparative genomic hybridisation. aCGH results previously reported, Natrajan et al. [9]., Hunngermann et al. [30], and Turner et al. [19]. Histological grade assessed by the modified Scarff-Bloom-Richardson system [32]. [file bcr3154-S1.PDF]
