# Supplementary figures and images for: Functional characterization of the 19q12 amplicon in grade III breast cancers
Source: Breast Cancer Res. 2012 Mar 20;14(2):R53. doi: 10.1186/bcr3154 (PMC3446387; doi:10.1186/bcr3154)

# Supplementary Figure 2

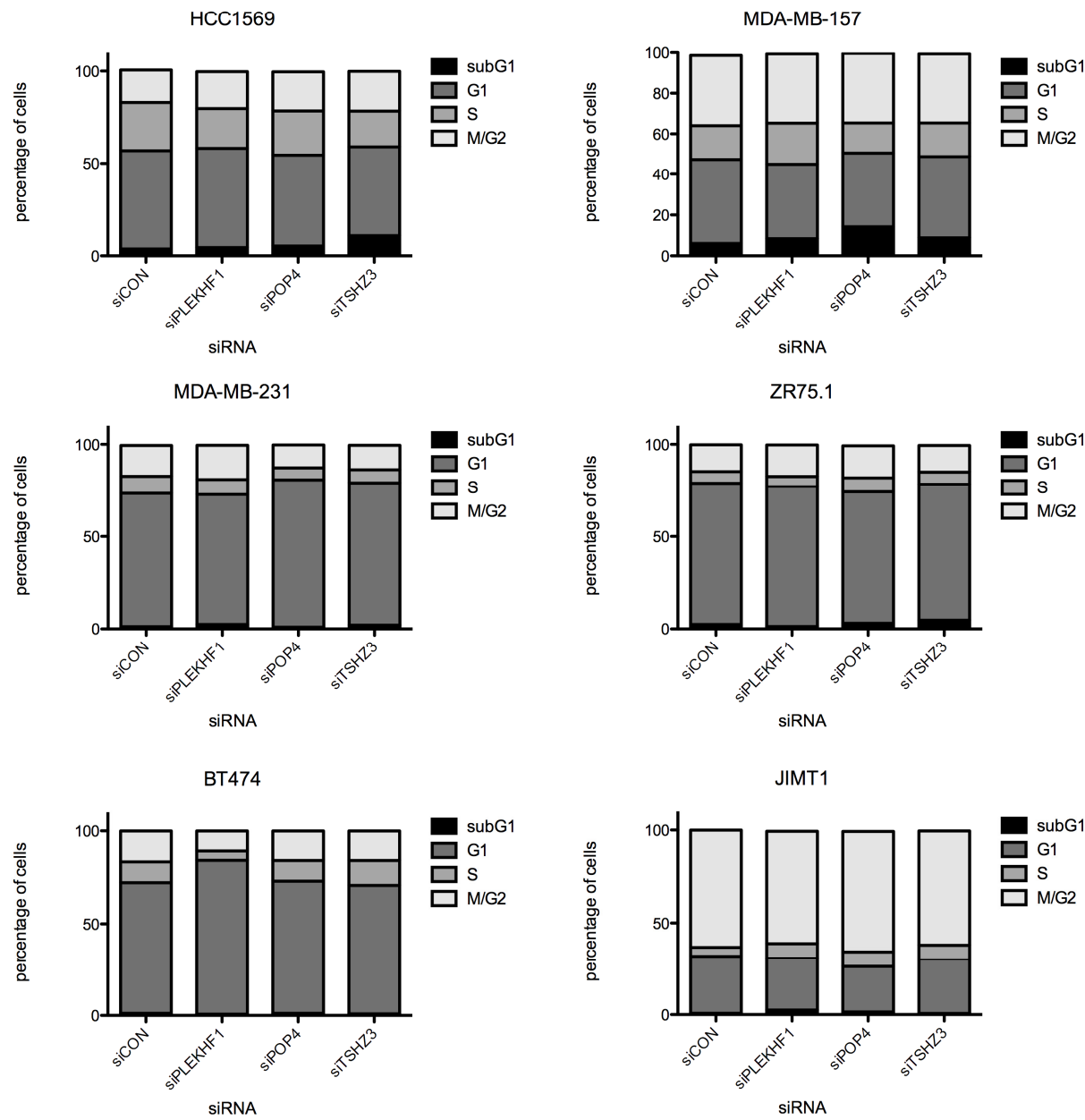

Supplement: Additional file 2 — Table S2. Details of the 56 breast cancer cell lines subjected to microarray-based comparative genomic hybridisation. ER status, HER2 gene amplification status and p53 protein levels and mutational status (adapted from Neve et. al. [38], Arriola et al. [20] and [69] *p53 mRNA levels derived from Mackay et al. [16]. p53 protein levels and mutational status (obtained from COSMIC [70] and Neve et al. [38]). "Positive", detectable protein expression; MUT, mutant; "Negative", no detectable protein expression; WT, wild-type. [file bcr3154-S2.PDF]

# Supplementary Figure 3

A

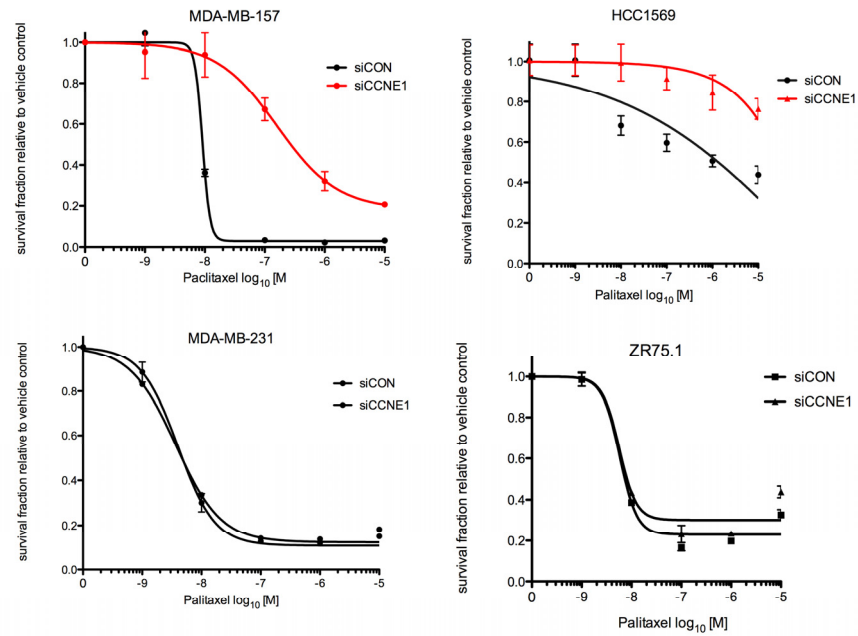

B

i

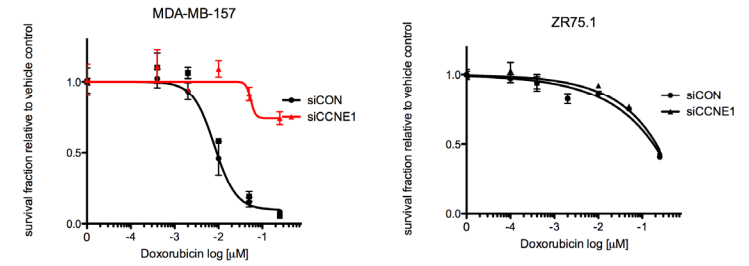

ii

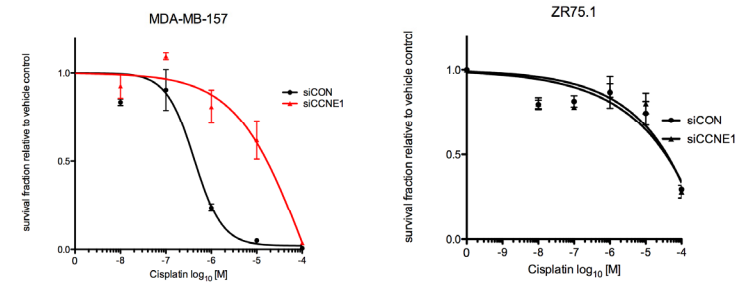

iii

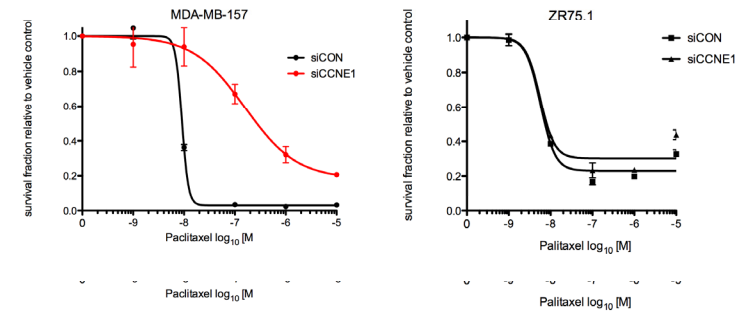

Supplement: Additional file 3 — Figure S1. Correlation of aCGH smoothed Log2ratios and FISH. Microarray-CGH chromosome 19 plots illustrating the patterns of 19q12 amplification in two primary breast cancers (bright green, circular binary segmentation (cbs)-smoothed Log2 ratio > 0.45), copy number gain in two primary breast cancers (green, circular binary segmentation (cbs)-smoothed Log2 ratio > 0.08 to ≤ 0.45 and no copy number change in two primary breast cancers (black, circular binary segmentation (cbs)-smoothed Log2 ratio > -0.08 to < 0.08). Log2 ratios (x-axis) are plotted against BAC position (y-axis). Red: loss, green: gain, bright green: amplification, purple line: smoothed cbs ratios; amp: 19q12 amplified, gain: 19q12 copy number gain, no change: 19q12 no copy number change. Fluorescence in situ hybridisation (FISH) confirmation of 19q12 copy number status with a biotin-labelled BAC probe (RP11-327I05, mapping to 30.10 to 30.25 Mb, red). Top two cases show amplification with > 5 copies per nucleus, middle two cases low level gain with three to five copies per nucleus, and bottom two cases no copy number change with two copies per nucleus. [file bcr3154-S3.PDF]
