# Supplementary material for: Functional characterization of the 19q12 amplicon in grade III breast cancers
Source: Breast Cancer Res. 2012 Mar 20;14(2):R53. doi: 10.1186/bcr3154 (PMC3446387; doi:10.1186/bcr3154)
Supplement: Additional file 4 — Figure S2. Effect of RNA interference gene silencing on cell cycle profiles. Cell cycle profiles of cancer cells harbouring 19q12 amplification (that is, MDA-MB-157 and HCC1569) or lacking amplification of this locus (MDA-MB-231, JIMT1, ZR75.1 and BT474) after RNAi silencing of POP4, PLEKHF1 and TSHZ3. Note the slight increase in the fraction of subG1 in amplified cells, but no difference in the proportion of G1 or S/G2 between cell lines with or without amplification at 19q12. [file bcr3154-S4.PDF]

Sample Identifier	Grade	ER	PR	HER2	Subtype	19q12 amp	CCNE1 amp	PLEKHF1 amp	TP53 mutation	Reference
B34	3	Negative	Negative	Amplified	HER2-positive	Amplified	Amplified	Amplified	c.484A>C, p.I162L; Missense	Natrajan et al. - Reference 9
B41	3	Negative	Negative	Not amplified	ER-negative/HER2-negative	Amplified	Amplified	Amplified	c.709A>G, p.M237V; Misense	Natrajan et al. - Reference 9
NKI-22	3	Negative	Negative	Not amplified	ER-negative/HER2-negative	Amplified	Amplified	Amplified	c.375+2T>C; Splice	Not previously published
NKI-91	3	Negative	Negative	Not amplified	ER-negative/HER2-negative	Amplified	Amplified	Amplified	c.673-2A>C; Splice	Not previously published
TN.7880	3	Negative	Negative	Not amplified	ER-negative/HER2-negative	Amplified	Amplified	Amplified	c.659A>G, p.Y220C; Missense	Turner et al. - Reference 19
B21	3	Positive	Positive	Not amplified	ER-positive/HER2-negative	Amplified	Not amplified	Not amplified	no mutation	Natrajan et al. - Reference 9
B30	3	Positive	Positive	Not amplified	ER-positive/HER2-negative	Amplified	Not amplified	Not amplified	no mutation	Natrajan et al. - Reference 9
B6	3	Negative	Negative	Not amplified	ER-negative/HER2-negative	Amplified	Not amplified	Not amplified	c.524G>A,p.R175H; Missense	Natrajan et al. - Reference 9
BC2968	3	Negative	Negative	Not amplified	ER-negative/HER2-negative	Amplified	Not amplified	Amplified	c.586C>T,p.R196X; Nonsense	Natrajan et al. - Reference 9
NKI-16	3	Negative	Negative	Not amplified	ER-negative/HER2-negative	Amplified	Not amplified	Not amplified	c.527G>C, p.C176S; Missense	Not previously published
NKI-27	3	Positive	Negative	Not amplified	ER-positive/HER2-negative	Amplified	Not amplified	Not amplified	no mutation	Not previously published
NKI-37	3	Negative	Negative	Not amplified	ER-negative/HER2-negative	Amplified	Not amplified	Not amplified	c.488A>G, p.Y163C; Missense	Not previously published
NKI-40	3	Negative	Negative	Not amplified	ER-negative/HER2-negative	Amplified	Not amplified	Not amplified	c.217delG; c.997delC; Frameshift	Not previously published
NKI-63	3	Negative	Negative	Amplified	HER2-positive	Amplified	Not amplified	Not amplified	no mutation	Not previously published
NT0015	3	Negative	Negative	Not amplified	ER-negative/HER2-negative	Amplified	Not amplified	Amplified	c.722C>T,p.S241F; Missense	Not previously published
NT005	3	Negative	Negative	Not amplified	ER-negative/HER2-negative	Amplified	Not amplified	Amplified	c.709A>A,p.M237V; Missense	Not previously published
B1	3	Positive	Negative	Not amplified	ER-positive/HER2-negative	Not amplified	Not amplified	Not amplified	NA	Natrajan et al. - Reference 9
B10	3	Negative	Negative	Amplified	HER2-positive	Not amplified	Not amplified	Not amplified	NA	Natrajan et al. - Reference 9
B11	3	Negative	Positive	Not amplified	ER-negative/HER2-negative	Not amplified	Not amplified	Not amplified	NA	Natrajan et al. - Reference 9
B12	3	Negative	Negative	Amplified	HER2-positive	Not amplified	Not amplified	Not amplified	NA	Natrajan et al. - Reference 9
B13	2	Negative	Positive	Amplified	HER2-positive	Not amplified	Not amplified	Not amplified	NA	Natrajan et al. - Reference 9
B15	3	Positive	Positive	Not amplified	ER-positive/HER2-negative	Not amplified	Not amplified	Not amplified	NA	Natrajan et al. - Reference 9
B16	3	Positive	Positive	Not amplified	ER-positive/HER2-negative	Not amplified	Not amplified	Not amplified	NA	Natrajan et al. - Reference 9
B17	3	Negative	Negative	Not amplified	ER-negative/HER2-negative	Not amplified	Not amplified	Not amplified	NA	Natrajan et al. - Reference 9
B2	3	Negative	Negative	Not amplified	ER-negative/HER2-negative	Not amplified	Not amplified	Not amplified	NA	Natrajan et al. - Reference 9
B20	3	Negative	Negative	Amplified	HER2-positive	Not amplified	Not amplified	Not amplified	NA	Natrajan et al. - Reference 9
B22	2	Positive	Positive	Amplified	HER2-positive	Not amplified	Not amplified	Not amplified	NA	Natrajan et al. - Reference 9
B24	3	Positive	Negative	Not amplified	ER-positive/HER2-negative	Not amplified	Not amplified	Not amplified	NA	Natrajan et al. - Reference 9
B25	2	Negative	Negative	Amplified	HER2-positive	Not amplified	Not amplified	Not amplified	NA	Natrajan et al. - Reference 9
B26	3	Positive	Negative	Amplified	HER2-positive	Not amplified	Not amplified	Not amplified	NA	Natrajan et al. - Reference 9
B27	3	Positive	Positive	Not amplified	ER-positive/HER2-negative	Not amplified	Not amplified	Not amplified	NA	Natrajan et al. - Reference 9
B29	3	Negative	Negative	Not amplified	ER-negative/HER2-negative	Not amplified	Not amplified	Not amplified	NA	Natrajan et al. - Reference 9
B3	3	Positive	Positive	Not amplified	ER-positive/HER2-negative	Not amplified	Not amplified	Not amplified	NA	Natrajan et al. - Reference 9
B31	3	Positive	Positive	Not amplified	ER-positive/HER2-negative	Not amplified	Not amplified	Not amplified	NA	Natrajan et al. - Reference 9
B32	3	Negative	Negative	Amplified	HER2-positive	Not amplified	Not amplified	Not amplified	NA	Natrajan et al. - Reference 9
B33	3	Positive	Positive	Not amplified	ER-positive/HER2-negative	Not amplified	Not amplified	Not amplified	NA	Natrajan et al. - Reference 9
B35	3	Negative	Negative	Amplified	HER2-positive	Not amplified	Not amplified	Not amplified	NA	Natrajan et al. - Reference 9
B36	3	Positive	Positive	Not amplified	ER-positive/HER2-negative	Not amplified	Not amplified	Not amplified	NA	Natrajan et al. - Reference 9
B37	3	Positive	Positive	Not amplified	ER-positive/HER2-negative	Not amplified	Not amplified	Not amplified	NA	Natrajan et al. - Reference 9
B38	3	Positive	Negative	Not amplified	ER-positive/HER2-negative	Not amplified	Not amplified	Not amplified	NA	Natrajan et al. - Reference 9
B39	3	Positive	Positive	Not amplified	ER-positive/HER2-negative	Not amplified	Not amplified	Not amplified	NA	Natrajan et al. - Reference 9
B4	2	Positive	Negative	Amplified	HER2-positive	Not amplified	Not amplified	Not amplified	NA	Natrajan et al. - Reference 9
B40	3	Negative	Positive	Amplified	HER2-positive	Not amplified	Not amplified	Not amplified	NA	Natrajan et al. - Reference 9
B43	3	Negative	Negative	Amplified	HER2-positive	Not amplified	Not amplified	Not amplified	NA	Natrajan et al. - Reference 9
B44	3	Positive	Positive	Not amplified	ER-positive/HER2-negative	Not amplified	Not amplified	Not amplified	NA	Natrajan et al. - Reference 9
B45	3	Negative	Negative	Not amplified	ER-negative/HER2-negative	Not amplified	Not amplified	Not amplified	NA	Natrajan et al. - Reference 9
B46	3	Negative	Negative	Amplified	HER2-positive	Not amplified	Not amplified	Not amplified	NA	Natrajan et al. - Reference 9
B47	3	Positive	Negative	Not amplified	ER-positive/HER2-negative	Not amplified	Not amplified	Not amplified	NA	Natrajan et al. - Reference 9
B49	3	Positive	Positive	Not amplified	ER-positive/HER2-negative	Not amplified	Not amplified	Not amplified	NA	Natrajan et al. - Reference 9
B5	3	Positive	Positive	Not amplified	ER-positive/HER2-negative	Not amplified	Not amplified	Not amplified	NA	Natrajan et al. - Reference 9
B50	3	Positive	Positive	Not amplified	ER-positive/HER2-negative	Not amplified	Not amplified	Not amplified	NA	Natrajan et al. - Reference 9
B52	3	Negative	Negative	Amplified	HER2-positive	Not amplified	Not amplified	Not amplified	NA	Natrajan et al. - Reference 9
B53	3	Negative	Negative	Not amplified	ER-negative/HER2-negative	Not amplified	Not amplified	Not amplified	NA	Natrajan et al. - Reference 9
B55	3	Positive	Positive	Not amplified	ER-positive/HER2-negative	Not amplified	Not amplified	Not amplified	NA	Natrajan et al. - Reference 9
B58	3	Positive	Positive	Not amplified	ER-positive/HER2-negative	Not amplified	Not amplified	Not amplified	NA	Natrajan et al. - Reference 9
B60	3	Positive	Positive	Not amplified	ER-positive/HER2-negative	Not amplified	Not amplified	Not amplified	NA	Natrajan et al. - Reference 9
B61	3	Positive	Positive	Not amplified	ER-positive/HER2-negative	Not amplified	Not amplified	Not amplified	NA	Natrajan et al. - Reference 9
B62	3	Negative	Negative	Not amplified	ER-negative/HER2-negative	Not amplified	Not amplified	Not amplified	NA	Natrajan et al. - Reference 9
B64	3	Negative	Negative	Not amplified	ER-negative/HER2-negative	Not amplified	Not amplified	Not amplified	NA	Natrajan et al. - Reference 9
B65	3	Negative	Negative	Not amplified	ER-negative/HER2-negative	Not amplified	Not amplified	Not amplified	NA	Natrajan et al. - Reference 9
B66	3	Negative	Negative	Amplified	HER2-positive	Not amplified	Not amplified	Not amplified	NA	Natrajan et al. - Reference 9
B67	3	Negative	Negative	Not amplified	ER-negative/HER2-negative	Not amplified	Not amplified	Not amplified	NA	Natrajan et al. - Reference 9
B69	3	Negative	Negative	Not amplified	ER-negative/HER2-negative	Not amplified	Not amplified	Not amplified	NA	Natrajan et al. - Reference 9
B7	3	Positive	Negative	Not amplified	ER-positive/HER2-negative	Not amplified	Not amplified	Not amplified	NA	Natrajan et al. - Reference 9
B71	3	Negative	Negative	Amplified	HER2-positive	Not amplified	Not amplified	Not amplified	NA	Natrajan et al. - Reference 9
B72	3	Positive	Positive	Not amplified	ER-positive/HER2-negative	Not amplified	Not amplified	Not amplified	NA	Natrajan et al. - Reference 9
B73	3	Negative	Negative	Not amplified	ER-negative/HER2-negative	Not amplified	Not amplified	Not amplified	NA	Natrajan et al. - Reference 9
B74	3	Positive	Negative	Not amplified	ER-positive/HER2-negative	Not amplified	Not amplified	Not amplified	NA	Natrajan et al. - Reference 9
B76	3	Positive	Positive	Amplified	HER2-positive	Not amplified	Not amplified	Not amplified	NA	Natrajan et al. - Reference 9
B77	3	Positive	Positive	Amplified	HER2-positive	Not amplified	Not amplified	Not amplified	NA	Natrajan et al. - Reference 9
B78	3	Negative	Negative	Not amplified	ER-negative/HER2-negative	Not amplified	Not amplified	Not amplified	NA	Natrajan et al. - Reference 9
B79	3	Negative	Negative	Not amplified	ER-negative/HER2-negative	Not amplified	Not amplified	Not amplified	NA	Natrajan et al. - Reference 9
B8	3	Positive	Positive	Not amplified	ER-positive/HER2-negative	Not amplified	Not amplified	Not amplified	NA	Natrajan et al. - Reference 9
B80	3	Negative	Negative	Amplified	HER2-positive	Not amplified	Not amplified	Not amplified	NA	Natrajan et al. - Reference 9
B81	3	Negative	Negative	Not amplified	ER-negative/HER2-negative	Not amplified	Not amplified	Not amplified	NA	Natrajan et al. - Reference 9
B82	3	Positive	Positive	Not amplified	ER-positive/HER2-negative	Not amplified	Not amplified	Not amplified	NA	Natrajan et al. - Reference 9
B83	3	Negative	Negative	Amplified	HER2-positive	Not amplified	Not amplified	Not amplified	NA	Natrajan et al. - Reference 9
B84	3	Negative	Negative	Not amplified	ER-negative/HER2-negative	Not amplified	Not amplified	Not amplified	NA	Natrajan et al. - Reference 9
B86	3	Negative	Negative	Not amplified	ER-negative/HER2-negative	Not amplified	Not amplified	Not amplified	NA	Natrajan et al. - Reference 9
B87	3	Negative	Negative	Not amplified	ER-negative/HER2-negative	Not amplified	Not amplified	Not amplified	NA	Natrajan et al. - Reference 9
B9	3	Positive	Negative	Not amplified	ER-positive/HER2-negative	Not amplified	Not amplified	Not amplified	NA	Natrajan et al. - Reference 9
BC181	2	Positive	Positive	Not amplified	ER-positive/HER2-negative	Not amplified	Not amplified	Not amplified	NA	Hungermann et al. - Reference 31
BC184	3	Negative	Negative	Not amplified	ER-negative/HER2-negative	Not amplified	Not amplified	Not amplified	NA	Natrajan et al. - Reference 9
BC185	2	Positive	Negative	Amplified	HER2-positive	Not amplified	Not amplified	Not amplified	NA	Hungermann et al. - Reference 31
BC1921	3	Positive	Positive	Not amplified	ER-positive/HER2-negative	Not amplified	Not amplified	Not amplified	NA	Natrajan et al. - Reference 9
BC2048	2	Positive	Positive	Not amplified	ER-positive/HER2-negative	Not amplified	Not amplified	Not amplified	NA	Not previously published
BC2049	2	Positive	Positive	Amplified	HER2-positive	Not amplified	Not amplified	Not amplified	NA	Not previously published
BC2050	2	Positive	Positive	Not amplified	ER-positive/HER2-negative	Not amplified	Not amplified	Not amplified	NA	Not previously published
BC2052	1	Positive	Positive	Not amplified	ER-positive/HER2-negative	Not amplified	Not amplified	Not amplified	NA	Not previously published
BC2053	2	Positive	Positive	Not amplified	ER-positive/HER2-negative	Not amplified	Not amplified	Not amplified	NA	Not previously published
BC2054	1	Positive	Positive	Not amplified	ER-positive/HER2-negative	Not amplified	Not amplified	Not amplified	NA	Not previously published
BC2056	2	Positive	Positive	Not amplified	ER-positive/HER2-negative	Not amplified	Not amplified	Not amplified	NA	Not previously published
BC2062	3	Positive	Positive	Not amplified	ER-positive/HER2-negative	Not amplified	Not amplified	Not amplified	NA	Not previously published
BC2064	1	Positive	Positive	Not amplified	ER-positive/HER2-negative	Not amplified	Not amplified	Not amplified	NA	Not previously published
BC2065	2	Positive	Positive	Not amplified	ER-positive/HER2-negative	Not amplified	Not amplified	Not amplified	NA	Not previously published
BC2066	2	Positive	Positive	Not amplified	ER-positive/HER2-negative	Not amplified	Not amplified	Not amplified	NA	Not previously published
BC2067	2	Positive	Positive	Not amplified	ER-positive/HER2-negative	Not amplified	Not amplified	Not amplified	NA	Not previously published
BC2068	2	Positive	Positive	Not amplified	ER-positive/HER2-negative	Not amplified	Not amplified	Not amplified	NA	Not previously published
BC2071	1	Positive	Positive	Not amplified	ER-positive/HER2-negative	Not amplified	Not amplified	Not amplified	NA	Not previously published
BC2072	1	Positive	Positive	Not amplified	ER-positive/HER2-negative	Not amplified	Not amplified	Not amplified	NA	Not previously published
BC2468	2	Positive	Positive	Not amplified	ER-positive/HER2-negative	Not amplified	Not amplified	Not amplified	NA	Hungermann et al. - Reference 31
BC2469	2	Positive	Positive	Not amplified	ER-positive/HER2-negative	Not amplified	Not amplified	Not amplified	NA	Not previously published
BC2471	2	Positive	Positive	Not amplified	ER-positive/HER2-negative	Not amplified	Not amplified	Not amplified	NA	Not previously published
BC2472	2	Positive	Positive	Not amplified	ER-positive/HER2-negative	Not amplified	Not amplified	Not amplified	NA	Hungermann et al. - Reference 31
BC2473	2	Positive	Positive	Not amplified	ER-positive/HER2-negative	Not amplified	Not amplified	Not amplified	NA	Not previously published
BC2511	3	Negative	Negative	Amplified	HER2-positive	Not amplified	Not amplified	Not amplified	NA	Natrajan et al. - Reference 9
BC2512	2	Positive	Negative	Not amplified	ER-positive/HER2-negative	Not amplified	Not amplified	Not amplified	NA	Not previously published
BC2517	2	Positive	Positive	Not amplified	ER-positive/HER2-negative	Not amplified	Not amplified	Not amplified	NA	Hungermann et al. - Reference 31
BC2519	3	Positive	Positive	Not amplified	ER-positive/HER2-negative	Not amplified	Not amplified	Not amplified	NA	Natrajan et al. - Reference 9
BC2522	2	Positive	Positive	Not amplified	ER-positive/HER2-negative	Not amplified	Not amplified	Not amplified	NA	Not previously published
BC2523	3	Positive	Positive	Not amplified	ER-positive/HER2-negative	Not amplified	Not amplified	Not amplified	NA	Natrajan et al. - Reference 9
BC2527	3	Negative	Negative	Not amplified	ER-negative/HER2-negative	Not amplified	Not amplified	Not amplified	NA	Not previously published
BC2528	3	Negative	Negative	Amplified	HER2-positive	Not amplified	Not amplified	Not amplified	NA	Natrajan et al. - Reference 9
BC2529	3	Positive	Negative	Not amplified	ER-positive/HER2-negative	Not amplified	Not amplified	Not amplified	NA	Natrajan et al. - Reference 9
BC2697	3	Negative	Negative	Not amplified	ER-negative/HER2-negative	Not amplified	Not amplified	Not amplified	NA	Natrajan et al. - Reference 9
BC2698	2	Positive	Positive	Not amplified	ER-positive/HER2-negative	Not amplified	Not amplified	Not amplified	NA	Not previously published
BC2700	2	Positive	Positive	Not amplified	ER-positive/HER2-negative	Not amplified	Not amplified	Not amplified	NA	Hungermann et al. - Reference 31
BC2703	2	Positive	Positive	Not amplified	ER-positive/HER2-negative	Not amplified	Not amplified	Not amplified	NA	Not previously published
BC2704	2	Positive	Positive	Not amplified	ER-positive/HER2-negative	Not amplified	Not amplified	Not amplified	NA	Hungermann et al. - Reference 31
BC2964	3	Positive	Positive	Not amplified	ER-positive/HER2-negative	Not amplified	Not amplified	Not amplified	NA	Natrajan et al. - Reference 9

BC2965	3	Positive	Positive	Not amplified	ER-positive/HER2-negative	Not amplified	Not amplified	Not amplified	NA	Natrajan et al. - Reference 9
BC2970	3	Negative	Negative	Amplified	HER2-positive	Not amplified	Not amplified	Not amplified	NA	Natrajan et al. - Reference 9
BC2973	2	Positive	Positive	Not amplified	ER-positive/HER2-negative	Not amplified	Not amplified	Not amplified	NA	Not previously published
BC2979	2	Positive	Positive	Not amplified	ER-positive/HER2-negative	Not amplified	Not amplified	Not amplified	NA	Not previously published
BC2982	3	Negative	Negative	Amplified	HER2-positive	Not amplified	Not amplified	Not amplified	NA	Natrajan et al. - Reference 9
BC3010	2	Positive	Positive	Not amplified	ER-positive/HER2-negative	Not amplified	Not amplified	Not amplified	NA	Not previously published
BC3013	2	Positive	Positive	Not amplified	ER-positive/HER2-negative	Not amplified	Not amplified	Not amplified	NA	Not previously published
BC3014	2	Positive	Positive	Not amplified	ER-positive/HER2-negative	Not amplified	Not amplified	Not amplified	NA	Not previously published
BC3015	2	Positive	Positive	Not amplified	ER-positive/HER2-negative	Not amplified	Not amplified	Not amplified	NA	Not previously published
BC3017	2	Positive	Positive	Not amplified	ER-positive/HER2-negative	Not amplified	Not amplified	Not amplified	NA	Not previously published
BC3036	3	Positive	Positive	Not amplified	ER-positive/HER2-negative	Not amplified	Not amplified	Not amplified	NA	Natrajan et al. - Reference 9
BC3038	3	Positive	Positive	Not amplified	ER-positive/HER2-negative	Not amplified	Not amplified	Not amplified	NA	Natrajan et al. - Reference 9
BC3041	3	Negative	Negative	Not amplified	ER-negative/HER2-negative	Not amplified	Not amplified	Not amplified	NA	Hungermann et al. - Reference 31
BC3042	3	Positive	Positive	Not amplified	ER-positive/HER2-negative	Not amplified	Not amplified	Not amplified	NA	Not previously published
BC3044	3	Negative	Negative	Not amplified	ER-negative/HER2-negative	Not amplified	Not amplified	Not amplified	NA	Natrajan et al. - Reference 9
BC3045	2	Negative	Negative	Not amplified	ER-negative/HER2-negative	Not amplified	Not amplified	Not amplified	NA	Not previously published
BC3046	2	Positive	Positive	Not amplified	ER-positive/HER2-negative	Not amplified	Not amplified	Not amplified	NA	Not previously published
BC3047	2	Negative	Negative	Not amplified	ER-negative/HER2-negative	Not amplified	Not amplified	Not amplified	NA	Not previously published
BT540	1	Positive	NA	Not amplified	ER-positive/HER2-negative	Not amplified	Not amplified	Not amplified	NA	Not previously published
BT544	3	Positive	NA	Amplified	HER2-positive	Not amplified	Not amplified	Not amplified	NA	Not previously published
BT673	1	Positive	NA	Not amplified	ER-positive/HER2-negative	Not amplified	Not amplified	Not amplified	NA	Not previously published
NKI-1	2	Positive	Positive	Not amplified	ER-positive/HER2-negative	Not amplified	Not amplified	Not amplified	NA	Not previously published
NKI-10	3	Negative	Negative	Not amplified	ER-negative/HER2-negative	Not amplified	Not amplified	Not amplified	NA	Not previously published
NKI-100	3	Negative	Negative	Not amplified	ER-negative/HER2-negative	Not amplified	Not amplified	Not amplified	NA	Not previously published
NKI-101	1	Positive	Positive	Not amplified	ER-positive/HER2-negative	Not amplified	Not amplified	Not amplified	NA	Not previously published
NKI-102	3	Positive	Positive	Not amplified	ER-positive/HER2-negative	Not amplified	Not amplified	Not amplified	NA	Not previously published
NKI-103	2	Positive	NA	Not amplified	ER-positive/HER2-negative	Not amplified	Not amplified	Not amplified	NA	Not previously published
NKI-104	2	Positive	NA	Not amplified	ER-positive/HER2-negative	Not amplified	Not amplified	Not amplified	NA	Not previously published
NKI-105	3	Positive	NA	Amplified	HER2-positive	Not amplified	Not amplified	Not amplified	NA	Not previously published
NKI-106	3	Negative	NA	Not amplified	ER-negative/HER2-negative	Not amplified	Not amplified	Not amplified	NA	Not previously published
NKI-107	3	Negative	NA	Not amplified	ER-negative/HER2-negative	Not amplified	Not amplified	Not amplified	NA	Not previously published
NKI-108	1	Positive	NA	Not amplified	ER-positive/HER2-negative	Not amplified	Not amplified	Not amplified	NA	Not previously published
NKI-109	1	Positive	NA	Not amplified	ER-positive/HER2-negative	Not amplified	Not amplified	Not amplified	NA	Not previously published
NKI-11	3	Negative	Negative	Not amplified	ER-negative/HER2-negative	Not amplified	Not amplified	Not amplified	NA	Not previously published
NKI-110	3	Negative	NA	Not amplified	ER-negative/HER2-negative	Not amplified	Not amplified	Not amplified	NA	Not previously published
NKI-111	2	Positive	NA	Not amplified	ER-positive/HER2-negative	Not amplified	Not amplified	Not amplified	NA	Not previously published
NKI-112	3	Positive	NA	Not amplified	ER-positive/HER2-negative	Not amplified	Not amplified	Not amplified	NA	Not previously published
NKI-113	3	Positive	NA	Amplified	HER2-positive	Not amplified	Not amplified	Not amplified	NA	Not previously published
NKI-114	3	Positive	NA	Not amplified	ER-positive/HER2-negative	Not amplified	Not amplified	Not amplified	NA	Not previously published
NKI-115	3	Negative	NA	Not amplified	ER-negative/HER2-negative	Not amplified	Not amplified	Not amplified	NA	Not previously published
NKI-116	2	Positive	NA	Not amplified	ER-positive/HER2-negative	Not amplified	Not amplified	Not amplified	NA	Not previously published
NKI-117	2	Positive	NA	Not amplified	ER-positive/HER2-negative	Not amplified	Not amplified	Not amplified	NA	Not previously published
NKI-118	1	Positive	NA	Not amplified	ER-positive/HER2-negative	Not amplified	Not amplified	Not amplified	NA	Not previously published
NKI-119	3	Positive	NA	Not amplified	ER-positive/HER2-negative	Not amplified	Not amplified	Not amplified	NA	Not previously published
NKI-12	3	Negative	Negative	Not amplified	ER-negative/HER2-negative	Not amplified	Not amplified	Not amplified	NA	Not previously published
NKI-120	3	Negative	NA	Not amplified	ER-negative/HER2-negative	Not amplified	Not amplified	Not amplified	NA	Not previously published
NKI-121	3	Negative	NA	Not amplified	ER-negative/HER2-negative	Not amplified	Not amplified	Not amplified	NA	Not previously published
NKI-122	2	Positive	NA	Not amplified	ER-positive/HER2-negative	Not amplified	Not amplified	Not amplified	NA	Not previously published
NKI-123	2	Positive	NA	Not amplified	ER-positive/HER2-negative	Not amplified	Not amplified	Not amplified	NA	Not previously published
NKI-124	3	Negative	NA	Not amplified	ER-negative/HER2-negative	Not amplified	Not amplified	Not amplified	NA	Not previously published
NKI-125	3	Negative	NA	Not amplified	ER-negative/HER2-negative	Not amplified	Not amplified	Not amplified	NA	Not previously published
NKI-126	3	Positive	NA	Not amplified	ER-positive/HER2-negative	Not amplified	Not amplified	Not amplified	NA	Not previously published
NKI-127	3	Negative	NA	Not amplified	ER-negative/HER2-negative	Not amplified	Not amplified	Not amplified	NA	Not previously published
NKI-128	2	Positive	NA	Not amplified	ER-positive/HER2-negative	Not amplified	Not amplified	Not amplified	NA	Not previously published
NKI-129	2	Positive	NA	Not amplified	ER-positive/HER2-negative	Not amplified	Not amplified	Not amplified	NA	Not previously published
NKI-13	3	Negative	Negative	Not amplified	ER-negative/HER2-negative	Not amplified	Not amplified	Not amplified	NA	Not previously published
NKI-130	3	Positive	NA	Not amplified	ER-positive/HER2-negative	Not amplified	Not amplified	Not amplified	NA	Not previously published
NKI-131	1	Positive	NA	Not amplified	ER-positive/HER2-negative	Not amplified	Not amplified	Not amplified	NA	Not previously published
NKI-132	2	Positive	NA	Not amplified	ER-positive/HER2-negative	Not amplified	Not amplified	Not amplified	NA	Not previously published
NKI-133	1	Positive	NA	Not amplified	ER-positive/HER2-negative	Not amplified	Not amplified	Not amplified	NA	Not previously published
NKI-134	3	Negative	NA	Not amplified	ER-negative/HER2-negative	Not amplified	Not amplified	Not amplified	NA	Not previously published
NKI-135	3	Positive	NA	Not amplified	ER-positive/HER2-negative	Not amplified	Not amplified	Not amplified	NA	Not previously published
NKI-136	1	Positive	NA	Not amplified	ER-positive/HER2-negative	Not amplified	Not amplified	Not amplified	NA	Not previously published
NKI-137	1	Positive	NA	Not amplified	ER-positive/HER2-negative	Not amplified	Not amplified	Not amplified	NA	Not previously published
NKI-138	2	Positive	NA	Not amplified	ER-positive/HER2-negative	Not amplified	Not amplified	Not amplified	NA	Not previously published
NKI-139	1	Positive	NA	Not amplified	ER-positive/HER2-negative	Not amplified	Not amplified	Not amplified	NA	Not previously published
NKI-14	3	Negative	Negative	Not amplified	ER-negative/HER2-negative	Not amplified	Not amplified	Not amplified	NA	Not previously published
NKI-140	1	Positive	NA	Not amplified	ER-positive/HER2-negative	Not amplified	Not amplified	Not amplified	NA	Not previously published
NKI-141	2	Positive	NA	Not amplified	ER-positive/HER2-negative	Not amplified	Not amplified	Not amplified	NA	Not previously published
NKI-142	2	Positive	NA	Not amplified	ER-positive/HER2-negative	Not amplified	Not amplified	Not amplified	NA	Not previously published
NKI-143	1	Positive	NA	Not amplified	ER-positive/HER2-negative	Not amplified	Not amplified	Not amplified	NA	Not previously published
NKI-144	2	Positive	NA	Not amplified	ER-positive/HER2-negative	Not amplified	Not amplified	Not amplified	NA	Not previously published
NKI-145	2	Positive	NA	Not amplified	ER-positive/HER2-negative	Not amplified	Not amplified	Not amplified	NA	Not previously published
NKI-146	2	Positive	NA	Not amplified	ER-positive/HER2-negative	Not amplified	Not amplified	Not amplified	NA	Not previously published
NKI-147	3	Negative	NA	Not amplified	ER-negative/HER2-negative	Not amplified	Not amplified	Not amplified	NA	Not previously published
NKI-148	2	Positive	NA	Not amplified	ER-positive/HER2-negative	Not amplified	Not amplified	Not amplified	NA	Not previously published
NKI-149	3	Positive	NA	Amplified	HER2-positive	Not amplified	Not amplified	Not amplified	NA	Not previously published
NKI-15	3	Negative	Negative	Not amplified	ER-negative/HER2-negative	Not amplified	Not amplified	Not amplified	NA	Not previously published
NKI-150	3	Positive	NA	Not amplified	ER-positive/HER2-negative	Not amplified	Not amplified	Not amplified	NA	Not previously published
NKI-17	3	Negative	Negative	Not amplified	ER-negative/HER2-negative	Not amplified	Not amplified	Not amplified	NA	Not previously published
NKI-18	3	Negative	Negative	Not amplified	ER-negative/HER2-negative	Not amplified	Not amplified	Not amplified	NA	Not previously published
NKI-19	3	Negative	Negative	Not amplified	ER-negative/HER2-negative	Not amplified	Not amplified	Not amplified	NA	Not previously published
NKI-2	3	Positive	Positive	Not amplified	ER-positive/HER2-negative	Not amplified	Not amplified	Not amplified	NA	Not previously published
NKI-20	3	Negative	Negative	Not amplified	ER-negative/HER2-negative	Not amplified	Not amplified	Not amplified	NA	Not previously published
NKI-21	3	Negative	Negative	Not amplified	ER-negative/HER2-negative	Not amplified	Not amplified	Not amplified	NA	Not previously published
NKI-23	3	Negative	Negative	Not amplified	ER-negative/HER2-negative	Not amplified	Not amplified	Not amplified	NA	Not previously published
NKI-24	1	Positive	Positive	Not amplified	ER-positive/HER2-negative	Not amplified	Not amplified	Not amplified	NA	Not previously published
NKI-25	1	Positive	Positive	Not amplified	ER-positive/HER2-negative	Not amplified	Not amplified	Not amplified	NA	Not previously published
NKI-26	1	Positive	Negative	Not amplified	ER-positive/HER2-negative	Not amplified	Not amplified	Not amplified	NA	Not previously published
NKI-28	1	Positive	Positive	Not amplified	ER-positive/HER2-negative	Not amplified	Not amplified	Not amplified	NA	Not previously published
NKI-29	2	Positive	Positive	Not amplified	ER-positive/HER2-negative	Not amplified	Not amplified	Not amplified	NA	Not previously published
NKI-3	2	Positive	Positive	Not amplified	ER-positive/HER2-negative	Not amplified	Not amplified	Not amplified	NA	Not previously published
NKI-30	1	Positive	Positive	Not amplified	ER-positive/HER2-negative	Not amplified	Not amplified	Not amplified	NA	Not previously published
NKI-31	1	Positive	Negative	Not amplified	ER-positive/HER2-negative	Not amplified	Not amplified	Not amplified	NA	Not previously published
NKI-32	1	Positive	Positive	Not amplified	ER-positive/HER2-negative	Not amplified	Not amplified	Not amplified	NA	Not previously published
NKI-33	2	Positive	Positive	Not amplified	ER-positive/HER2-negative	Not amplified	Not amplified	Not amplified	NA	Not previously published
NKI-34	2	Positive	Positive	Not amplified	ER-positive/HER2-negative	Not amplified	Not amplified	Not amplified	NA	Not previously published
NKI-35	1	Positive	Positive	Not amplified	ER-positive/HER2-negative	Not amplified	Not amplified	Not amplified	NA	Not previously published
NKI-36	3	Negative	Negative	Not amplified	ER-negative/HER2-negative	Not amplified	Not amplified	Not amplified	NA	Not previously published
NKI-38	3	Negative	Negative	Not amplified	ER-negative/HER2-negative	Not amplified	Not amplified	Not amplified	NA	Not previously published
NKI-39	2	Negative	Negative	Not amplified	ER-negative/HER2-negative	Not amplified	Not amplified	Not amplified	NA	Not previously published
NKI-4	3	Positive	Negative	Not amplified	ER-positive/HER2-negative	Not amplified	Not amplified	Not amplified	NA	Not previously published
NKI-41	2	Negative	Negative	Not amplified	ER-negative/HER2-negative	Not amplified	Not amplified	Not amplified	NA	Not previously published
NKI-42	1	Negative	Negative	Not amplified	ER-negative/HER2-negative	Not amplified	Not amplified	Not amplified	NA	Not previously published
NKI-43	1	Negative	Negative	Not amplified	ER-negative/HER2-negative	Not amplified	Not amplified	Not amplified	NA	Not previously published
NKI-44	2	Negative	Negative	Not amplified	ER-negative/HER2-negative	Not amplified	Not amplified	Not amplified	NA	Not previously published
NKI-45	1	Negative	Negative	Not amplified	ER-negative/HER2-negative	Not amplified	Not amplified	Not amplified	NA	Not previously published
NKI-46	3	Positive	Negative	Not amplified	ER-positive/HER2-negative	Not amplified	Not amplified	Not amplified	NA	Not previously published
NKI-47	2	Negative	Negative	Not amplified	ER-negative/HER2-negative	Not amplified	Not amplified	Not amplified	NA	Not previously published
NKI-48	2	Positive	Negative	Not amplified	ER-positive/HER2-negative	Not amplified	Not amplified	Not amplified	NA	Not previously published
NKI-49	2	Negative	Negative	Not amplified	ER-negative/HER2-negative	Not amplified	Not amplified	Not amplified	NA	Not previously published
NKI-5	2	Positive	Positive	Not amplified	ER-positive/HER2-negative	Not amplified	Not amplified	Not amplified	NA	Not previously published
NKI-50	1	Positive	Positive	Not amplified	ER-positive/HER2-negative	Not amplified	Not amplified	Not amplified	NA	Not previously published
NKI-51	2	Positive	Positive	Not amplified	ER-positive/HER2-negative	Not amplified	Not amplified	Not amplified	NA	Not previously published
NKI-52	2	Positive	Positive	Not amplified	ER-positive/HER2-negative	Not amplified	Not amplified	Not amplified	NA	Not previously published
NKI-53	2	Positive	Positive	Not amplified	ER-positive/HER2-negative	Not amplified	Not amplified	Not amplified	NA	Not previously published
NKI-54	2	Positive	Positive	Not amplified	ER-positive/HER2-negative	Not amplified	Not amplified	Not amplified	NA	Not previously published
NKI-55	2	Positive	Negative	Not amplified	ER-positive/HER2-negative	Not amplified	Not amplified	Not amplified	NA	Not previously published
NKI-56	2	Positive	Positive	Not amplified	ER-positive/HER2-negative	Not amplified	Not amplified	Not amplified	NA	Not previously published
NKI-57	3	Negative	Negative	Not amplified	ER-negative/HER2-negative	Not amplified	Not amplified	Not amplified	NA	Not previously published
NKI-58	3	Positive	Negative	Not amplified	ER-positive/HER2-negative	Not amplified	Not amplified	Not amplified	NA	Not previously published

NKI-59	3	Positive	Positive	Not amplified	ER-positive/HER2-negative	Not amplified	Not amplified	Not amplified	NA	Not previously published
NKI-6	2	Positive	Negative	Not amplified	ER-positive/HER2-negative	Not amplified	Not amplified	Not amplified	NA	Not previously published
NKI-60	3	Negative	Negative	Amplified	HER2-positive	Not amplified	Not amplified	Not amplified	NA	Not previously published
NKI-61	3	Negative	Negative	Amplified	HER2-positive	Not amplified	Not amplified	Not amplified	NA	Not previously published
NKI-62	3	Negative	Negative	Amplified	HER2-positive	Not amplified	Not amplified	Not amplified	NA	Not previously published
NKI-64	3	Negative	Negative	Amplified	HER2-positive	Not amplified	Not amplified	Not amplified	NA	Not previously published
NKI-65	3	Negative	Negative	Amplified	HER2-positive	Not amplified	Not amplified	Not amplified	NA	Not previously published
NKI-66	3	Negative	Negative	Amplified	HER2-positive	Not amplified	Not amplified	Not amplified	NA	Not previously published
NKI-67	3	Negative	Negative	Amplified	HER2-positive	Not amplified	Not amplified	Not amplified	NA	Not previously published
NKI-68	3	Negative	Negative	Amplified	HER2-positive	Not amplified	Not amplified	Not amplified	NA	Not previously published
NKI-69	3	Negative	Negative	Amplified	HER2-positive	Not amplified	Not amplified	Not amplified	NA	Not previously published
NKI-7	2	Positive	Negative	Not amplified	ER-positive/HER2-negative	Not amplified	Not amplified	Not amplified	NA	Not previously published
NKI-70	3	Positive	Negative	Amplified	HER2-positive	Not amplified	Not amplified	Not amplified	NA	Not previously published
NKI-71	3	Negative	Negative	Amplified	HER2-positive	Not amplified	Not amplified	Not amplified	NA	Not previously published
NKI-72	3	Positive	Negative	Amplified	HER2-positive	Not amplified	Not amplified	Not amplified	NA	Not previously published
NKI-73	3	Positive	Negative	Amplified	HER2-positive	Not amplified	Not amplified	Not amplified	NA	Not previously published
NKI-74	3	Positive	Positive	Amplified	HER2-positive	Not amplified	Not amplified	Not amplified	NA	Not previously published
NKI-75	2	Negative	Negative	Amplified	HER2-positive	Not amplified	Not amplified	Not amplified	NA	Not previously published
NKI-76	3	Negative	NA	Amplified	HER2-positive	Not amplified	Not amplified	Not amplified	NA	Not previously published
NKI-77	3	Negative	Negative	Amplified	HER2-positive	Not amplified	Not amplified	Not amplified	NA	Not previously published
NKI-78	3	Negative	Negative	Amplified	HER2-positive	Not amplified	Not amplified	Not amplified	NA	Not previously published
NKI-79	3	Negative	Negative	Amplified	HER2-positive	Not amplified	Not amplified	Not amplified	NA	Not previously published
NKI-8	3	Negative	Negative	Amplified	HER2-positive	Not amplified	Not amplified	Not amplified	NA	Not previously published
NKI-80	3	Negative	Negative	Amplified	HER2-positive	Not amplified	Not amplified	Not amplified	NA	Not previously published
NKI-81	3	Negative	Negative	Amplified	HER2-positive	Not amplified	Not amplified	Not amplified	NA	Not previously published
NKI-82	3	Positive	Negative	Amplified	HER2-positive	Not amplified	Not amplified	Not amplified	NA	Not previously published
NKI-83	3	Negative	Negative	Amplified	HER2-positive	Not amplified	Not amplified	Not amplified	NA	Not previously published
NKI-84	3	Negative	Negative	Amplified	HER2-positive	Not amplified	Not amplified	Not amplified	NA	Not previously published
NKI-85	3	Positive	Positive	Amplified	HER2-positive	Not amplified	Not amplified	Not amplified	NA	Not previously published
NKI-86	3	Negative	Negative	Amplified	HER2-positive	Not amplified	Not amplified	Not amplified	NA	Not previously published
NKI-87	3	Positive	Negative	Amplified	HER2-positive	Not amplified	Not amplified	Not amplified	NA	Not previously published
NKI-88	3	Positive	Negative	Amplified	HER2-positive	Not amplified	Not amplified	Not amplified	NA	Not previously published
NKI-89	3	Positive	Positive	Amplified	HER2-positive	Not amplified	Not amplified	Not amplified	NA	Not previously published
NKI-9	1	Positive	Positive	Not amplified	ER-positive/HER2-negative	Not amplified	Not amplified	Not amplified	NA	Not previously published
NKI-90	3	Negative	Negative	Amplified	HER2-positive	Not amplified	Not amplified	Not amplified	NA	Not previously published
NKI-92	3	Negative	Negative	Not amplified	ER-negative/HER2-negative	Not amplified	Not amplified	Not amplified	NA	Not previously published
NKI-93	3	Negative	Negative	Not amplified	ER-negative/HER2-negative	Not amplified	Not amplified	Not amplified	NA	Not previously published
NKI-94	2	Positive	Positive	Not amplified	ER-positive/HER2-negative	Not amplified	Not amplified	Not amplified	NA	Not previously published
NKI-95	3	Positive	Positive	Not amplified	ER-positive/HER2-negative	Not amplified	Not amplified	Not amplified	NA	Not previously published
NKI-96	1	Positive	Positive	Not amplified	ER-positive/HER2-negative	Not amplified	Not amplified	Not amplified	NA	Not previously published
NKI-97	3	Positive	Negative	Not amplified	ER-positive/HER2-negative	Not amplified	Not amplified	Not amplified	NA	Not previously published
NKI-98	3	Positive	Positive	Not amplified	ER-positive/HER2-negative	Not amplified	Not amplified	Not amplified	NA	Not previously published
NKI-99	2	Positive	Positive	Not amplified	ER-positive/HER2-negative	Not amplified	Not amplified	Not amplified	NA	Not previously published
NT0001	3	Negative	Negative	Not amplified	ER-negative/HER2-negative	Not amplified	Not amplified	Not amplified	NA	Not previously published
NT0017	3	Negative	Negative	Amplified	HER2-positive	Not amplified	Not amplified	Not amplified	NA	Not previously published
NT0024	3	Negative	Negative	Not amplified	ER-negative/HER2-negative	Not amplified	Not amplified	Not amplified	NA	Not previously published
NT0039	3	Negative	Negative	Not amplified	ER-negative/HER2-negative	Not amplified	Not amplified	Not amplified	NA	Not previously published
NT004	3	Negative	Negative	Not amplified	ER-negative/HER2-negative	Not amplified	Not amplified	Not amplified	NA	Not previously published
NT0059	3	Negative	Negative	Not amplified	ER-negative/HER2-negative	Not amplified	Not amplified	Not amplified	NA	Not previously published
NT0067	3	Negative	Negative	Not amplified	ER-negative/HER2-negative	Not amplified	Not amplified	Not amplified	NA	Not previously published
TN.10030	3	Negative	Negative	Not amplified	ER-negative/HER2-negative	Not amplified	Not amplified	Not amplified	NA	Turner et al. - Reference 19
TN.10137	3	Negative	Negative	Not amplified	ER-negative/HER2-negative	Not amplified	Not amplified	Not amplified	NA	Turner et al. - Reference 19
TN.10391	3	Negative	Negative	Not amplified	ER-negative/HER2-negative	Not amplified	Not amplified	Not amplified	NA	Turner et al. - Reference 19
TN.10499	3	Negative	Negative	Not amplified	ER-negative/HER2-negative	Not amplified	Not amplified	Not amplified	NA	Turner et al. - Reference 19
TN.10646	3	Negative	Negative	Not amplified	ER-negative/HER2-negative	Not amplified	Not amplified	Not amplified	NA	Turner et al. - Reference 19
TN.10662	3	Negative	Negative	Not amplified	ER-negative/HER2-negative	Not amplified	Not amplified	Not amplified	NA	Turner et al. - Reference 19
TN.10665	3	Negative	Negative	Not amplified	ER-negative/HER2-negative	Not amplified	Not amplified	Not amplified	NA	Turner et al. - Reference 19
TN.10752	3	Negative	Negative	Not amplified	ER-negative/HER2-negative	Not amplified	Not amplified	Not amplified	NA	Turner et al. - Reference 19
TN.11093	3	Negative	Negative	Not amplified	ER-negative/HER2-negative	Not amplified	Not amplified	Not amplified	NA	Turner et al. - Reference 19
TN.11155	3	Negative	Negative	Not amplified	ER-negative/HER2-negative	Not amplified	Not amplified	Not amplified	NA	Turner et al. - Reference 19
TN.12303	3	Negative	Negative	Not amplified	ER-negative/HER2-negative	Not amplified	Not amplified	Not amplified	NA	Turner et al. - Reference 19
TN.12523	3	Negative	Negative	Not amplified	ER-negative/HER2-negative	Not amplified	Not amplified	Not amplified	NA	Turner et al. - Reference 19
TN.1267	3	Negative	Negative	Not amplified	ER-negative/HER2-negative	Not amplified	Not amplified	Not amplified	NA	Turner et al. - Reference 19
TN.1878	3	Negative	Negative	Not amplified	ER-negative/HER2-negative	Not amplified	Not amplified	Not amplified	NA	Turner et al. - Reference 19
TN.3319	3	Negative	Negative	Not amplified	ER-negative/HER2-negative	Not amplified	Not amplified	Not amplified	NA	Turner et al. - Reference 19
TN.3701	3	Negative	Negative	Not amplified	ER-negative/HER2-negative	Not amplified	Not amplified	Not amplified	NA	Turner et al. - Reference 19
TN.4171	3	Negative	Negative	Not amplified	ER-negative/HER2-negative	Not amplified	Not amplified	Not amplified	NA	Turner et al. - Reference 19
TN.4274	3	Negative	Negative	Not amplified	ER-negative/HER2-negative	Not amplified	Not amplified	Not amplified	NA	Turner et al. - Reference 19
TN.4643	3	Negative	Negative	Not amplified	ER-negative/HER2-negative	Not amplified	Not amplified	Not amplified	NA	Turner et al. - Reference 19
TN.5508	3	Negative	Negative	Not amplified	ER-negative/HER2-negative	Not amplified	Not amplified	Not amplified	NA	Turner et al. - Reference 19
TN.9138	3	Negative	Negative	Not amplified	ER-negative/HER2-negative	Not amplified	Not amplified	Not amplified	NA	Turner et al. - Reference 19
TN.9776	3	Negative	Negative	Not amplified	ER-negative/HER2-negative	Not amplified	Not amplified	Not amplified	NA	Turner et al. - Reference 19
