# Supplementary material for: Functional characterization of the 19q12 amplicon in grade III breast cancers
Source: Breast Cancer Res. 2012 Mar 20;14(2):R53. doi: 10.1186/bcr3154 (PMC3446387; doi:10.1186/bcr3154)
Supplement: Additional file 5 — Figure S3. CCNE1 gene silencing confers resistance to conventional chemotherapy agents. (A) Dose response curves following treatment with paclitaxel after CCNE1 silencing using short hairpin RNAs in cancer cells harbouring CCNE1 gene amplification (that is, MDA-MB-157 and HCC1569) or lacking amplification at this locus (that is, MDA-MB-231 and ZR75.1). (B) Dose response curves after CCNE1 silencing to i) doxorubicin, ii) cisplatin and iii) paclitaxel in cancer cells harbouring (MDA-MB-157) or lacking (ZR75.1) 19q12 amplification. [file bcr3154-S5.PDF]

Supplementary Table 2. Details of the 56 breast cancer cell lines subjected to microarray-based comparative genomic hybridisation.

Cell line	ER	HER2	TP53	19q
HCC1569	Negative	Amplified	Negative/MUT	Amplified
MDA-MB-157	Negative	Not amplified	Negative/MUT	Amplified
BT20	Negative	Not amplified	Positive/ WT	Not amplified
BT474	Positive	Amplified	Positive/ MUT	Not amplified
BT483	Positive	Not amplified	Negative/MUT	Not amplified
BT549	Negative	Not amplified	Positive/ MUT	Not amplified
CAL120	Negative	Not amplified	MUT	Not amplified
CAL51	Negative	Not amplified	Positive*	Not amplified
CAMA1	Positive	Not amplified	Positive/MUT	Not amplified
DU4475	Negative	Not amplified	WT	Not amplified
GI101	Positive	Not amplified	Positive*	Not amplified
HB4A	Negative	Not amplified	Positive*	Not amplified
HBL100	Negative	Not amplified	Positive/WT	Not amplified
HCC1143	Negative	Not amplified	Positive/ MUT	Not amplified
HCC1187	Negative	Not amplified	Positive/ MUT	Not amplified
HCC1428	Positive	Not amplified	Positive	Not amplified
HCC1500	Negative	Not amplified	Negative	Not amplified
HCC1937	Negative	Not amplified	Negative/MUT	Not amplified
HCC1954	Negative	Amplified	Negative/MUT	Not amplified
HCC202	Negative	Amplified	Negative	Not amplified
HCC3153	Negative	Not amplified	Negative	Not amplified
HCC38	Negative	Not amplified	Positive/ MUT	Not amplified
HCC70	Negative	Not amplified	Positive/ MUT	Not amplified
HMT3552	Negative	Not amplified	Negative*	Not amplified
HS578T	Negative	Not amplified	Positive/ MUT	Not amplified
JIMT1	Negative	Amplified	NA	Not amplified
MCF10A	Negative	Not amplified	Negative/ WT	Not amplified
MCF12A	Negative	Not amplified	Positive/WT	Not amplified
MCF7	Positive	Not amplified	Negative/ WT	Not amplified
MDA-MB-134	Positive	Not amplified	Negative/ WT	Not amplified
MDA-MB-175	Positive	Not amplified	Negative/ WT	Not amplified
MDA-MB-231	Negative	Not amplified	Positive/ MUT	Not amplified
MDA-MB-361	Positive	Amplified	Negative/ WT	Not amplified
MDA-MB-436	Negative	Not amplified	Negative/MUT	Not amplified
MDA-MB-468	Negative	Not amplified	MUT	Not amplified
MDA-MB-469	Negative	Not amplified	Negative*	Not amplified
MFM223	Negative	Not amplified	MUT	Not amplified
PMC42	Negative	Not amplified	Positive*	Not amplified
S68	Positive	Not amplified	NA	Not amplified
SKBR3	Negative	Amplified	Positive/MUT	Not amplified
SKBR5	Negative	Not amplified	MUT	Not amplified
SKBR7	Negative	Not amplified	WT	Not amplified
SUM1315MO2	Negative	Not amplified	Positive	Not amplified
SUM149PT	Negative	Not amplified	Positive/MUT	Not amplified
SUM159PT	Negative	Not amplified	Negative/MUT	Not amplified
SUM190PT	Negative	Amplified	Negative/MUT	Not amplified
SUM225CWN	Negative	Amplified	Positive/MUT	Not amplified
SUM44PE	Positive	Not amplified	Negative/MUT	Not amplified
SUM52PE	Positive	Not amplified	Negative/MUT	Not amplified
T47D	Positive	Not amplified	Positive/ MUT	Not amplified
UACC3199	Negative	Not amplified	Positive*	Not amplified
UACC812	Positive	Amplified	Negative/ WT	Not amplified
UACC893	Negative	Amplified	MUT	Not amplified
VP229	Negative	Amplified	MUT	Not amplified
ZR-75.1	Positive	Not amplified	Negative/WT	Not amplified
ZR-75.30	Positive	Amplified	Negative/WT	Not amplified

ER status, HER2 gene amplification status and p53 protein levels and mutational status (adapted from Neve et. al. [39], Arriola et al. [20] and http://p53.free.fr/Database/Cancer_cell_lines/Breast_cancer.html). *p53 mRNA levels derived from Mackay et al. [16]. p53 protein levels and mutational status (obtained from COSMIC [66] and Neve et al. [39]). "Positive", detectable protein expression; MUT, mutant; "Negative", no detectable protein expression; WT, wild-type.
